# Inhibition of key enzymes linked to type 2 diabetes by compounds isolated from *Aframomum melegueta* fruit

**DOI:** 10.1080/13880209.2017.1286358

**Published:** 2017-02-08

**Authors:** Aminu Mohammed, Victoria Awolola Gbonjubola, Neil Anthony Koorbanally, Md. Shahidul Islam

**Affiliations:** a Department of Biochemistry, School of Life Sciences, University of KwaZulu-Natal, Durban, South Africa;; b Department of Biochemistry, Faculty of Science, Ahmadu Bello University, Zaria, Nigeria;; c Department of Chemistry, School of Chemistry and Physics, University of KwaZulu-Natal, Durban, South Africa

**Keywords:** Antidiabetic, α-amylase, α-glucosidase, kinetics

## Abstract

**Context:** The use of *Aframomum melegueta* K. Schum. (Zingiberaceae) fruit for treatment of diabetes has recently been established in Nigeria. However, compounds responsible for the antidiabetic action have not been identified.

**Objective:** The present study carried out the bioassay-guided isolation of possible bioactive compounds responsible for the antidiabetic action of *A. melegueta* fruit.

**Materials and methods:** The *A. melegueta* fruit was sequentially extracted using ethyl acetate (EtOAc), ethanol and water, and the most active extract (EtOAc) was subjected to column chromatography on a silica gel column using solvent gradient systems of hexane (HEX):EtOAc and EtOAc:MeOH and the isolation of compounds was guided by α-glycosidase and α-amylase inhibitory activities at various concentrations (30–240 μg/mL).

**Results:** According to the results, 3 arylalkanes, 6-paradol (**1**), 6-shogaol (**2**) and 6-gingerol (**3**) and a pentacyclic triterpene, oleanolic acid (**4**) were isolated from *A. melegueta* fruit. All the compounds exhibited inhibitory effects against α-amylase and α-glucosidase. 6-Gingerol (**3**) and oleanolic acid (**4**) showed higher inhibitory activity against α-amylase (IC_50_: 6-gingerol: 81.78 ± 7.79 μM; oleanolic acid: 91.72 ± 1.63 μM) and α-glucosidase (IC_50_: 6-gingerol: 21.55 ± 0.45 μM; oleanolic acid: 17.35 ± 0.88 μM) compared to the standard drug, acarbose and other isolated compounds. The kinetics of the enzyme action of the compounds showed a noncompetitive mode of inhibition.

**Conclusion:** The data of this study suggest that the 6-gingerol (**3**) and oleanolic acid (**4**) showed higher α-amylase and α-glucosidase inhibitory action and therefore could be responsible for the antidiabetic activity of *A. melegueta* fruit.

## Introduction

Type 2 diabetes (T2D) is a metabolic disorder characterized by chronic insulin resistance and loss of functional pancreatic β-cells due to relative (rather than absolute) deficiency of insulin (ADA 2015). The inability of the pancreatic β-cell to secrete enough insulin disrupts the regulation of hepatic glucose synthesis, muscles glucose uptake and adipose tissue lipolysis. The consequence is postprandial hyperglycemia, which subsequently causes T2D (Gastaldelli 2011). This postprandial hyperglycemia must be controlled in order to prevent diabetic complications (Rahati et al. 2014). Among many therapeutic approaches to reduce postprandial hyperglycemia in T2D, delaying glucose absorption via inhibition of intestinal carbohydrate-digesting enzymes, such as α-glucosidase and α-amylase, has gained popularity in the recent years (Imam 2015). Acarbose and miglitol are the two major available glucosidase inhibitors currently used in the treatment of T2D. However, major drawbacks in using these existing drugs (acarbose and miglitol) are diarrhoea and abdominal pain (Irons & Minze 2014). Thus, the search for potent antidiabetic drugs with fewer side effects has been shifted to plant-based natural products (Cragg & Newman 2013). The use of plant-derived extracts and/or compounds as an alternative to synthetic drugs is preferred, as they possess fewer side-effects (Taveira et al. 2010).

*Aframomum melegueta* K. Schum. (Zingiberaceae) commonly known as guinea or alligator pepper is abundantly found in central and western parts of Africa (Iwu 2014). The fruit, leaf and seeds are used as flavouring agents in various traditional foods and for the treatment of diseases including diabetes and other related disorders in many parts of Africa (Abo et al. 2008; Gbolade 2009). Previous studies have shown that *A. melegueta* has hepato-protective (Nwozo & Oyinloye 2011), anti-inflammatory (Ilic et al. 2014) and antioxidative (Onoja et al. 2014) effects at *in vitro* and *in vivo* conditions. In a recent study, the diterpenoids isolated from the organic seed extract of *A. melegueta* showed bactericidal activity *in vitro* against *Escherichia coli, Listeria moncyogenes* and *Staphylococcus aureus* strains (Ngwoke et al. 2014). In another study, 6-paradol (**1**), 6-shogaol (**2**) and 6-gingerol (**3**) isolated from the seed have shown anti-inflammatory activity in a rat paw oedema model (Ilic et al. 2014). El-Halawany et al. (2014) have also reported that diarylheptanoid, 8-dehydrogingerdione, 6-dehydroparadol, dihydrogingerenone, 6-gingerol, dihydroparadol, 6-paradol and 6-shogaol demonstrated hepato-protective and antioxidative actions in CCl_4_ induced acute liver injury in rats.

Furthermore, some previous studies have reported that various extracts from *A. melegueta* have demonstrated inhibitory effects against α-glucosidase and α-amylase actions (Etoundi et al. 2010; Adefegha & Oboh 2011, 2012; Kazeem et al. 2012) which has been supported by our recent study (Mohammed et al. 2016). The seed and leaf extracts were also reported to demonstrate blood glucose lowering ability in alloxan-induced diabetic rats (Adesokan et al. 2010; Mojekwu et al. 2011). Additionally, our recent findings have shown that the ethyl acetate (EtOAc) fraction derived from the crude ethanol extract exhibited inhibitory effects against α-glucosidase and α-amylase activities *in vitro* (Mohammed et al. 2016), anti-hyperglycemic and anti-hyperlipidemic actions in experimentally-induced animal model of T2D (Mohammed et al. 2015). They have also shown the ability to ameliorate β-cell dysfunction and other major diabetes-related complications in a T2D model of rats (Mohammed et al. 2015). However, the compounds responsible for the antidiabetic action remain elusive.

Hence, the present study aimed at carrying out a bioassay-guided isolation of possible bioactive compounds responsible for the antidiabetic action of the *A. melegueta* fruit and their inhibitory effects on enzymes linked to T2D.

## Materials and methods

### Materials

*Saccharomyces cerevisiae* α-glucosidase (E.C. 3.2.1.20), porcine pancreatic α-amylase (E.C. 3.2.1.1), *p*-nitrophenyl glucopyranoside (*p*NPG) and *p*-nitrophenol were obtained from Sigma-Aldrich through Capital Lab Supplies, New Germany, South Africa. Starch, dinitrosalicylic acid (DNS), maltose, absolute ethanol and ethyl acetate were obtained from Merck Chemical Company, Durban, South Africa.

### Plant material

The fruit of *A. melegueta* was freshly collected in December, 2012 from Ibadan, Oyo State, Nigeria and identified and authenticated at the herbarium unit of the Biological Science Department, Ahmadu Bello University, Zaria, Nigeria by Mr. Umar Gallah. A voucher specimen number 1511 was deposited accordingly. The sample was immediately washed and shade-dried for two weeks to constant weight. The dried sample was ground to a fine powder, and stored individually in airtight containers to transport to the University of KwaZulu-Natal, Westville Campus, Durban, South Africa for further investigations.

### Preparation of plant extracts

Extraction and fractionation were carried out according to the method reported previously (Ibrahim & Islam 2014). The *A. melegueta* EtOAc fraction was a dark greenish residue and demonstrated the highest *in vitro* α-glucosidase (IC_50_: 40.44 ± 5.77 μg/mL) and α-amylase (IC_50_: 68.69 ± 6.05 μg/mL) inhibitory activity amongst the fractions and thus selected for the isolation of possible bioactive compounds.

### Isolation of bioactive compounds from the EtOAc fraction

The EtOAc fraction (15.5 g) of *A. melegueta* fruit was subjected to column chromatography on a silica gel column using gradient solvent systems of hexane (HEX):EtOAc and EtOAc:MeOH with increments of 10% in each step to obtain 84 fractions. After monitoring with TLC, similar fractions were pooled together to obtain seven major fractions (A: 11–13; B: 14–17; C: 20–25; D: 26–32; E: 33–34; F: 35–47 and G: 48–55) that exhibited considerably higher inhibitory effects against α-amylase and α-glucosidase activities. Compound **1** (210.0 mg), was obtained as an oil from fraction C (2.51 g) after further purification on a column (2.0 cm diameter) using HEX:EtOAc (17:3) as an eluent. Fraction D (990.3 mg) was further purified on a column (1.5 cm in diameter) by eluting with HEX:EtOAc (8:2) to obtain compound **2** (490.0 mg) as an oil. Compound **3** (990.9 mg) was obtained as an oil by the purification of fraction F (4.13 g) on a silica column and eluted with HEX: EtOAc (3:2). Fraction E (100.9 mg) was further purified with a HEX:EtOAc (7:3) solvent system, which yielded compound **4** (60.0 mg). All isolated bioactive compounds demonstrated α-amylase and α-glucosidase inhibitory actions at different scale.

All NMR data, ^1^H, ^13^C and 2D experiments were recorded on a Bruker Avance III 400 MHz spectrometer. Samples were acquired with deuterated chloroform (CDCl_3_). The spectra were referenced according to the deuteriochloroform signal at δ_H_ 7.24 (for ^1^H NMR spectra) and δ_C_ 77.0 (for ^13^C NMR spectra) for CDCl_3_. The HRESIMS spectra were obtained on a Bruker Micro TOF-QII instrument (Coventry, UK).

### α*-*Amylase (E.C. 3.2.1.1) inhibitory effects

The α-amylase inhibitory effect of the compounds was carried out using a modified method of McCue and Shetty (2004). Briefly, a 250 μL aliquot of compounds at different concentrations (30, 60, 120 and 240 μg/mL) was placed in a test tube and 250 μL of 0.02 M sodium phosphate buffer (pH 6.9) containing α-amylase (2.0 U/mL) solution was added. This solution was pre-incubated at 25 °C for 10 min, after which 250 μL of 1% starch solution in 0.02 M sodium phosphate buffer (pH 6.9) was added at a time interval of 10 s and then further incubated at 25 °C for 10 min. The reaction was terminated after incubation by adding 1 mL of dinitrosalicylic acid (DNS) reagent. The tube was then boiled for 10 min and cooled to room temperature. The reaction mixture was diluted with 5 mL distilled water and the absorbance was measured at 540 nm using a Shimadzu UV mini 1240 spectrophotometer (Shimadzu Corporation, Kyoto, Japan). A control was prepared using the same procedure using distilled water instead of the compound.

The results of α-amylase inhibitory effects of compounds were expressed as a percentage of control (blank) according to the following formula:

% Inhibition = [(Abs of control−Abs of compound)/Abs of control] x 100.

### α*-*Glucosidase (E.C. 3.2.1.20) inhibitory effects

The inhibitory effect of the isolated compounds on α-glucosidase activity was determined according to the method described by Kim et al. (2005) using α-glucosidase from *Saccharomyces cerevisiae*. The 5.0 mM *p*-nitrophenyl glucopyranoside (*p*NPG) substrate solution was prepared in 20 mM phosphate buffer, pH 6.9. An aliquot of 500 μL of α-glucosidase (1.0 U/mL) was then pre-incubated with 250 μL of the different concentrations of compounds (30, 60, 120 and 240 μg/mL) for 10 min. Thereafter, 250 μL of 5.0 mM *p*NPG was dissolved in 20 mM phosphate buffer (pH 6.9) as a substrate to start the reaction. The reaction mixture was incubated at 37 °C for 30 min. The α-glucosidase activity was determined by measuring the yellow coloured *p*-nitrophenol released from *p*NPG at 405 nm.

The results of α-glucosidase inhibitory effects of compounds were expressed as a percentage of the control (blank) according to the following formula:

Inhibition=[(Abs of control-Abs of sample)/   Abs of control]×100.

### Mechanism of α-amylase and α-glucosidase inhibitions

The isolated compounds were subjected to kinetic experiments to determine the type of inhibition exerted on α-amylase and α-glucosidase. The experiment was conducted according to the protocols mentioned above at a constant concentration of the compounds (30 μg/mL) with a variable concentration of substrate. For the α-glucosidase inhibition assay, 0.313–5.0 mM *p*NPG and 0.063–1.0% starch was used. The initial rates of reactions were determined from calibration curves constructed using varying concentrations of *p*-nitrophenol (0.313–5.0 mM) and maltose (0.063–1.0%) for the α-glucosidase and α-amylase inhibition assays, respectively. The initial velocity data obtained were used to construct Lineweaver-Burke plots to determine the *K*_M_ (Michaelis constant) and *v*_max_ (maximum velocity) of the enzyme as well as the *K*i (inhibition binding constant as a measure of affinity of the inhibitor to the enzyme) and the type of inhibition for both enzymes.

### Statistical analysis

Data are presented as mean ± SD (*n* = 3). Data were analyzed by using a statistical software package (SPSS for Windows, version 18, IBM Corporation, NY) using Tukey’s-HSD multiple range *post hoc* test. Values were considered significantly different at *p* < 0.05.

## Results and discussion

**Figure 1.**
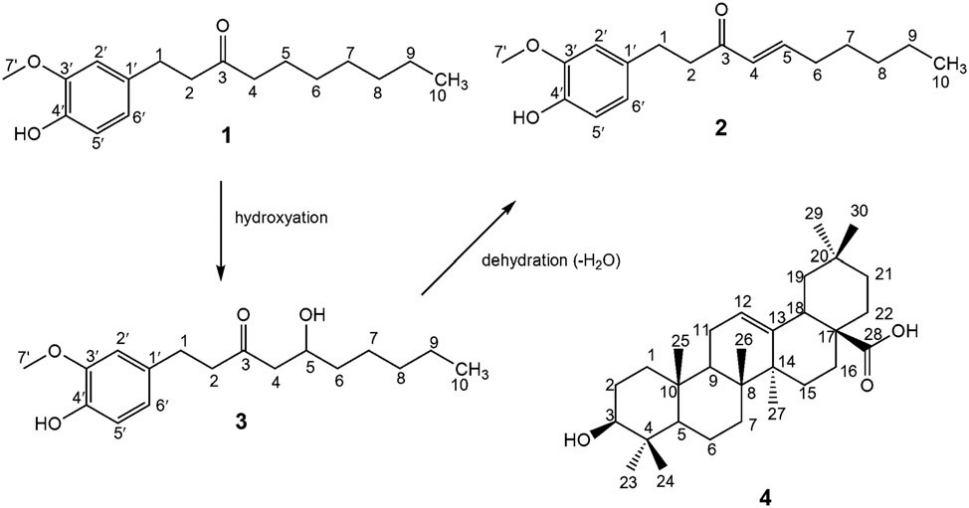
Structures of compounds **1–4** isolated from *A. melegueta*.

The phytochemical analysis of *A. melegueta* fruit led to the isolation of pure bioactive compounds **1–4** (Figure 1 and Table 1) which were characterized by 1D (^1^H and ^13^C) and 2D NMR and confirmed by comparison with data in the literature as 6-paradol (**1**) (Ma et al. 2004), 6-shogoal (**2**) and 6-gingerol (**3**) (Lee et al. 2011) and oleanolic acid (**4**) (Mahato & Kundu 1994). Compounds (**1–3**) are arylalkanes common to the Zingeberaceae. They all have a common 4-hydroxy-3-methoxyphenyl moiety and differ in the side chain due to the biosynthetic conversion of 6-paradol (**1**) to 6-gingerol (**3**) by hydroxylation and dehydration to 6-shogoal (**2**) (Figure 1). These compounds were previously isolated from *A. melegueta* seeds and reported to possess a variety of interesting pharmacological properties including hepato-protective and antioxidant activity (El-Halawany et al. 2014), as well as anti-inflammatory activity (Dugasani et al. 2010; Ilic et al. 2014). Furthermore, previous studies on the phytochemistry of *A. melegueta* indicated that this plant contains monoterpenoids, sesquiterpenoids and diterpenoids (Tane et al. 2005). However, in our present study, oleanolic acid (**4**), a pentacyclic triterpene, was isolated from *A. melegueta* fruit for the first time, which is not surprising since it is found quite commonly in the plant kingdom. Oleanolic acid (**4**) is a potent pharmacologically active compound having antioxidant, anti-inflammatory and antidiabetic activity (Pollier & Goossens 2012).

**Table 1.** ^1^H and ^13^C NMR data of compounds **1–4** isolated from *A. melegueta* (more details in the supplementary data file).

Pos.	**1** (^1^H)	**1** (^13^C)	**2** (^1^H)	**2** (^13^C)	**3** (^1^H)	**3** (^13^C)	Pos.	**4** (^1^H)	**4** (^13^C)
1	2.74–2.78 m	29.0	2.80–2.81 m	31.3	2.64–2.65 m	29.1	1	1.54–1.56 m	38.4
2	2.61–2.65 m	44.5	2.80–2.81 m	41.9	2.58–2.60 m	45.2	2	1.25–1.27 m	27.2
3		210.9		200.1		211.4	3	3.19 dd (*J* = 10.7, 4.1 Hz)	79.0
4	2.29–2.33 m	43.1	6.05 d (*J* = 15.9 Hz)	130.3	2.39–2.41 m	49.4	4		38.8
5	1.47–1.52 m	23.7	6.76–6.80 m	148.1	3.91–3.94 m	67.7	5	0.69 bs	55.2
6	1.19–1.25 m	31.6	2.12–2.16 m	32.4	1.13–1.34 m	36.6	6	1.29–1.32 m; 1.49–1.51 m	18.3
7	1.19–1.25 m	29.5	1.38–1.42 m	27.7	1.13–1.34 m	31.7	7	1.36–1.40 m	32.6
8	1.19–1.25 m	29.1	1.21–1.27 m	29.9	1.13–1.34 m	25.1	8		39.3
9	1.19–1.25 m	22.6	1.21–1.27 m	22.4	1.13–1.34 m	22.5	9	1.55–1.56 m	47.6
10	0.82 t (*J* = 6.8 Hz)	14.0	0.84 t (*J* = 6.7 Hz)	13.9	0.75 t (*J* = 6.8 Hz)	14.0	10		37.1
1′		133.0		133.1		132.6	11	1.94–1.96 m	22.9
2′	6.63 d (*J* = 1.8 Hz)	111.2	6.66 d (*J* = 1.8 Hz)	111.2	6.54 d (*J* = 1.8 Hz)	111.4	12	5.25 bs	122.6
3′		146.6		146.5		146.9	13		143.6
4′		144.0		143.9		144.0	14		41.6
5′	6.75 d (*J* = 8.0)	114.5	6.78 d (*J* = 8.1 Hz)	114.4	6.65 d (*J* = 8.0 Hz)	114.8	15	1.65–1.68 m; 1.02–1.08 m	27.7
6′	6.59 dd (*J* = 8.0, 1.8 Hz)	120.7	6.63 dd (*J* = 8.1, 1.8 Hz)	120.8	6.47 dd (*J* = 8.0, 1.8 Hz)	120.6	16	1.82–1.85 m; 1.05–1.07 m	23.4
7′	3.78 s	55.8	3.81 s	55.8	3.64 s	55.7	17		46.5
							18	2.79 dd (*J* = 13.5, 4.0 Hz)	41.0
							19	1.58–1.60 m	45.9
							20		30.7
							21	1.38–1.42 m	33.8
							22	1.72–1.76 m; 1.52–1.55 m	32.4
							23	0.95 s	28.1
							24	0.75 s	15.5
							25	0.89 s	15.3
							26	0.72 s	17.1
							27	1.10 s	25.9
							28		183.3
							29	0.88 s	33.1
							30	0.90 s	23.6

**Figure 2.**
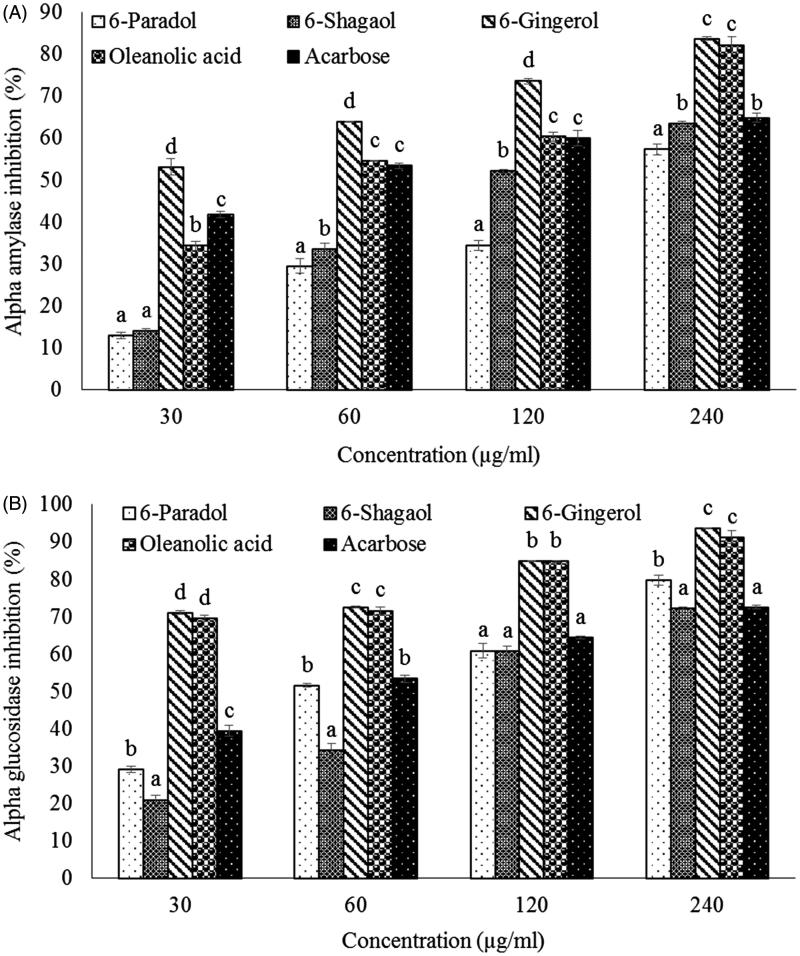
α-Amylase (A) and α-glucosidase (B) inhibitions (%) of compounds isolated from *A. melegueta* fruit. Data are presented as mean ± SD of triplicate determinations. a–e Values with different letters presented for a given concentration for each extract are significantly different from each other.

The data of the *in vitro* antidiabetic effect (inhibition of α-amylase and α-glucosidase activity) of the compounds from *A. melegueta* are presented in Figure 2 and Table 2 (IC_50_ values). It was observed that the IC_50_ values exhibited by oleanolic acid (**4**) (α-amylase: 91.72 ± 1.63 μM; α-glucosidase: 17.35 ± 0.88 μM) were significantly lower (*p <* 0.05) than the standard antidiabetic drug, acarbose and other isolated compounds (Table 2). According to some previous studies by de Melo et al. (2010), Ali et al. (2002) and Komaki et al. (2003), oleanolic acid (**4**) was shown to have an inhibitory effect on α-amylase (IC_50_: 154.89 μM) and α-glucosidase (IC_50_: 11.16 μM), respectively. This is in line with our present data and our study also additionally showed that oleanolic acid (**4**) is a potent inhibitor of α-glucosidase rather than α-amylase. It has been also reported that excessive inhibition of α-amylase action causes some undesirable consequences such as diarrhoea, bloating, flatulence, cramping and abdominal pain (Fujisawa et al. 2005). Hence, oleanolic acid (4) can be a better alternative to acarbose as an antidiabetic drug.

**Table 2.** IC_50_ values of bioactive compounds isolated from the *A. melegueta* fruit in antidiabetic models.

	IC_50_ (μM)	
Compounds	α-amylase inhibition	α-glucosidase inhibition
6-Paradol (**1**)	664.56 ± 11.58^d^	243.32 ± 6.65^d^
6-Shagaol (**2**)	443.17 ± 1.38^c^	326.11 ± 5.60^e^
6-Gingerol (**3**)	81.78 ± 7.79^a^	21.55 ± 0.45^b^
Oleanolic acid (**4**)	91.72 ± 1.63^b^	17.35 ± 0.88^a^
Acarbose	85.15 ± 5.56^a,b^	82.15 ± 2.42^c^

Data are presented as mean ± SD values of triplicate determinations.

^a–e^Different superscript letters presented within a column for a given parameter are significantly different from each other (Tukey’s-HSD multiple range *posthoc* test, *p <* 0.05).

Furthermore, the IC_50_ values observed with 6-gingerol (**3**) (α-amylase: 81.78 ± 7.79 μM; α-glucosidase: 21.55 ± 0.45 μM) were significantly lower (*p <* 0.05) compared to other two arylalkanes (Table 2). This shows that the C-5 hydroxyl group of the aliphatic side chain of 6-gingerol (**3**) contributed to the activity of this compound, however this is subject to further study. Moreover, the isolated arylalkanes (compounds **1–3**) have not been tested previously on α-amylase or α-glucosidase inhibitory actions, however, previous studies have shown that 6-shogoal (**2)** and 6-gingerol (**3**) play a crucial role in the treatment and control of T2D (Isa et al. 2008; Singh et al. 2009; Chakraborty et al. 2012; Shao et al. 2016). The lowest calculated IC_50_ values of compound **4** (α-amylase: 91.72 ± 1.63 μM; α-glucosidase: 17.35 ± 0.88 μM) and compound **3** (α-amylase: 81.78 ± 7.79 μM; α-glucosidase: 21.55 ± 0.45 μM) confirmed the excellent antidiabetic effects of *A. melegueta* fruit extract observed in our previous studies (Mohammed et al. 2015, 2016) which might be attributed to the presence of these compounds. Hence, 6-gingerol (**3**) and oleanolic acid (**4**) may be the most suitable antidiabetic agents compared to other isolated compounds. Additionally, a number of previous studies reported that 6-gingerol (**3**) and oleanolic acid (**4**) promote insulin secretion from pancreatic β-cells (Hsu et al. 2006; Teodoro et al. 2008; Lee et al. 2015). Therefore, the relatively elevated insulin levels observed in our previous study using the *A. melengueta* extract (Mohammed et al. 2015), could be attributed to the presence of some of these compounds as well.

More importantly, according to our previous data, the IC_50_ values of the compounds (**1–4**) were lower compared to that of crude ethanol extract (α-amylase: 0.62 ± 0.01 mg/mL; α-glucosidase: 0.06 ± 0.01 mg/mL), EtOAc fraction (α-amylase: 68.69 ± 6.05 μg/mL; α-glucosidase: 40.44 ± 5.77 μg/mL) (Mohammed et al. 2015, 2016) as well as other fractions (IC_50_ values >200 μg/mL) derived from the bioassay-guided analysis. This shows that further purification of the *A. melegueta* fruit extract leads to an increase α-amylase and α-glucosidase inhibitory effects. Therefore, the data of our present study indicate that the antidiabetic activity of *A. melegueta* fruit reported earlier (Adesokan et al. 2010; Mohammed et al. 2015, 2016) is attributed to the presence of isolated compounds (**1–4**).

**Figure 3.**
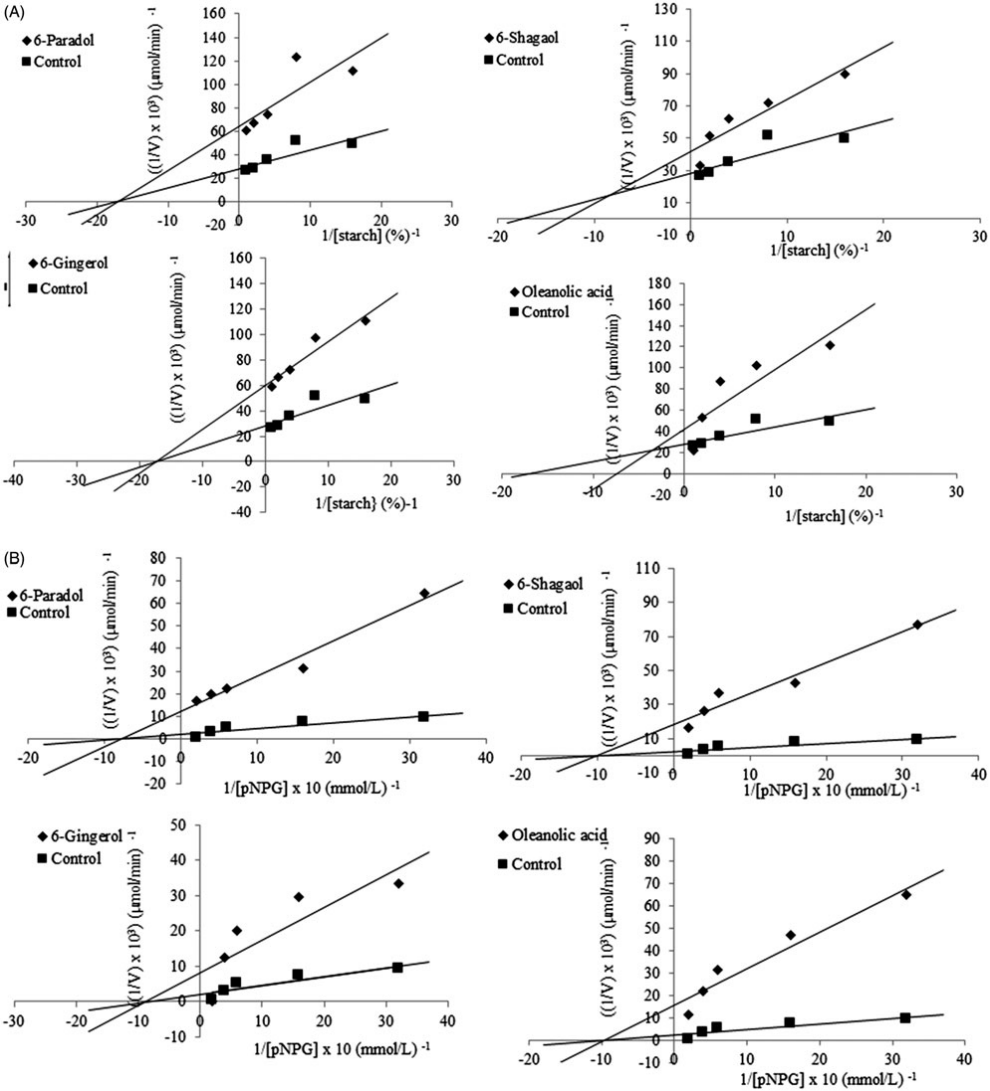
Lineweaver-Burke plots for *α*-amylase (A) and α-glucosidase (B) in the absence and presence of the inhibitors (bioactive compounds).

The kinetic data of the inhibitory mode of α-amylase and α-glucosidase enzymes by the isolated compounds are presented in Figure 3 and Table 3. The unchanged *K*_M_ and lower *v_max_* values denote the noncompetitive inhibition of α-amylase and α-glucosidase action by the compounds **1** and **3** (Figure 3, Table 3). Additionally, oleanolic acid (**4**) and 6-shogaol (**2**) showed a mixed inhibition type for α-amylase action where both the *K*_M_ and *v_max_* values were altered (Figure 3 and Table 3). It is therefore suggested that these compounds bind to other site(s), apart from the active site of the enzyme and induced a conformational change in the three-dimensional structure of the enzymes and ultimately slowed down their activities. The equilibrium constant for inhibitor binding (*K*_i_) of the compounds were lower for α-glucosidase than α-amylase, indicating greater stability of the enzyme-substrate complex for α-glucosidase compared to α-amylase enzyme (Table 3). This again confirms the potential of these compounds, particularly 6-gingerol (**3**) and oleanolic acid (**4**), for the development of novel and better antidiabetic drugs.

**Table 3.** Kinetic analysis of α-amylase and α-glucosidase inhibition by compounds isolated from the fruit of *A. melegueta* in antidiabetic models.

	α-Amylase inhibition			α-Glucosidase inhibition		
Compounds	*K_*M*_* (%)	*V_*max*_* (μmol/min)	*K_*i*_* (μg/mL)	*K_*M*_* (mM)	*V_*max*_* (μmol/min)	*K_*i*_* (μg/mL)
Control	0.06	35.65		1.10	471.56	
*A. melegueta*						
6-Paradol (**1**)	0.06	15.60	23.29	1.10	81.20	6.24
6-Shagaol (**2**)	0.09	24.10	62.33	1.10	55.70	4.02
6-Gingerol (**3**)	0.06	16.73	26.46	1.10	123.10	7.83
Oleanolic acid (**4**)	0.13	273.00	59.25	1.10	64.20	4.73

## Conclusions

In conclusion, the results of the present study indicate that 6-gingerol (**3**) and oleanolic acid (**4**) isolated from *A. melegueta* fruit demonstrate higher α-amylase and α-glucosidase inhibitory activity compared to other isolated compounds and could be regarded as possible antidiabetic agents. Therefore, the antidiabetic activity of the fruit of *A. melegueta* is attributed to the presence of these bioactive ingredients. Further clinical and detailed toxicological studies are required to fully confirm the potency of 6-gingerol (**3**) and oleanolic acid (**4**) as possible antidiabetic agents.

## Supplementary Material

Md_Shahidul_Islam_et_al_supplemental_content.zip
