# Supplementary material for: Inhibition of key enzymes linked to type 2 diabetes by compounds isolated from Aframomum melegueta fruit
Source: Pharm Biol. 2017 Feb 8;55(1):1010–6. doi: 10.1080/13880209.2017.1286358 (PMC6130490; doi:10.1080/13880209.2017.1286358)
Supplement: Md_Shahidul_Islam_et_al_supplemental_content.zip [file IPHB_A_1286358_SM5256.zip › Md Shahidul Islam et al supplemental content.pdf]

Supplementary data

Inhibition of key enzymes linked to type 2 diabetes by compounds isolated from *Aframomum melegueta* K. Schum. fruit

Aminu Mohammed[1,3], Gbonjubola Victoria Awolola[2], Neil Anthony Koorbanally[2] and Md. Shahidul Islam[1*]

[1]Department of Biochemistry, School of Life Sciences and [2]Department of Chemistry, School of Chemistry and Physics, University of KwaZulu-Natal, (Westville Campus), Durban, 4000, South Africa

[3]Department of Biochemistry, Faculty of Science, Ahmadu Bello University, Zaria-Nigeria

***Corresponding author:**

Prof. Md. Shahidul Islam

School of Life Sciences

University of KwaZulu-Natal (Westville Campus)

Durban 4000, South Africa.

Tel: +27 31 260 8717, Fax: +27 31 260 7942

Email: islamd@ukzn.ac.za or sislam1974@yahoo.com

For Peer Review Only

Contents

6-Paradol (**1**)

Figure S1. ^{1}H NMR spectrum of 6-paradol (**1**) ($CDCl_3$, 400 MHz).

Figure S2. ^{13}C NMR spectrum of 6-paradol (**1**) ($CDCl_3$, 400 MHz).

Figure S3. COSY NMR spectrum of 6-paradol (**1**)

Figure S4. DEPT spectrum of 6-paradol (**1**)

Figure S5. HMBC NMR spectrum of 6-paradol (**1**).

Figure S6. HSQC NMR spectrum of 6-paradol (**1**).

Figure S7. NOESY NMR spectrum 6-paradol (**1**).

6-Shagaol (**2**)

Figure S8. ^{1}H NMR spectrum of 6-shagaol (**2**) ($CDCl_3$, 400 MHz).

Figure S9. ^{13}C NMR spectrum of 6-shagaol (**2**) ($CDCl_3$, 400 MHz).

Figure S10. COSY NMR spectrum of 6-shagaol (**2**)

Figure S11. DEPT spectrum of 6-shagaol (**2**)

Figure S12. HMBC NMR spectrum of 6-shagaol (**2**).

Figure S13. HSQC NMR spectrum of 6-shagaol (**2**).

Figure S14. NOESY NMR spectrum 6-shagaol (**2**).

6-Gingerol (**3**)

Figure S15. ^{1}H NMR spectrum of 6-gingerol (**3**) ($CDCl_3$, 400 MHz).

Figure S16. ^{13}C NMR spectrum of 6-gingerol (**3**) ($CDCl_3$, 400 MHz).

Figure S17. COSY NMR spectrum of 6-gingerol (**3**)

Figure S18. DEPT spectrum of 6-gingerol (**3**)

Figure S19. HMBC NMR spectrum of 6-gingerol (**3**).

Figure S20. HSQC NMR spectrum of 6-gingerol (**3**).

Figure S21. NOESY NMR spectrum 6-gingerol (**3**).

Oleanolic acid (**4**)

Figure S22. ^{1}H NMR spectrum of oleanolic acid (**4**) ($CDCl_3$, 400 MHz).

For Peer Review Only

Figure S23. ^{13}C NMR spectrum of oleanolic acid (**4**) ($CDCl_3$, 400 MHz).

Figure S23. COSY NMR spectrum of oleanolic acid (**4**)

Figure S24. DEPT spectrum of oleanolic acid (**4**)

Figure S25. HMBC NMR spectrum of oleanolic acid (**4**).

Figure S26. HSQC NMR spectrum of oleanolic acid (**4**)

Figure S27. NOESY NMR spectrum oleanolic acid (**4**).

For Peer Re

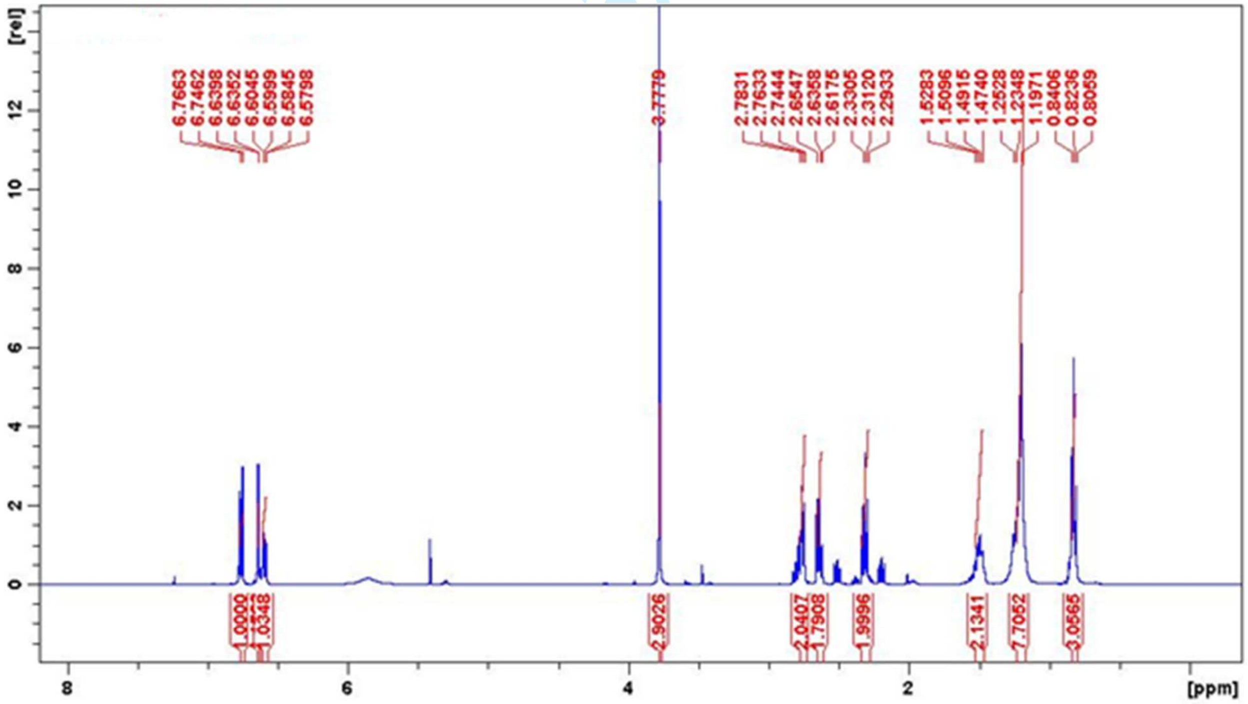

Figure S1: ^{1}H NMR spectrum of 6-paradol (**1**) ($CDCl_3$, 400 MHz).

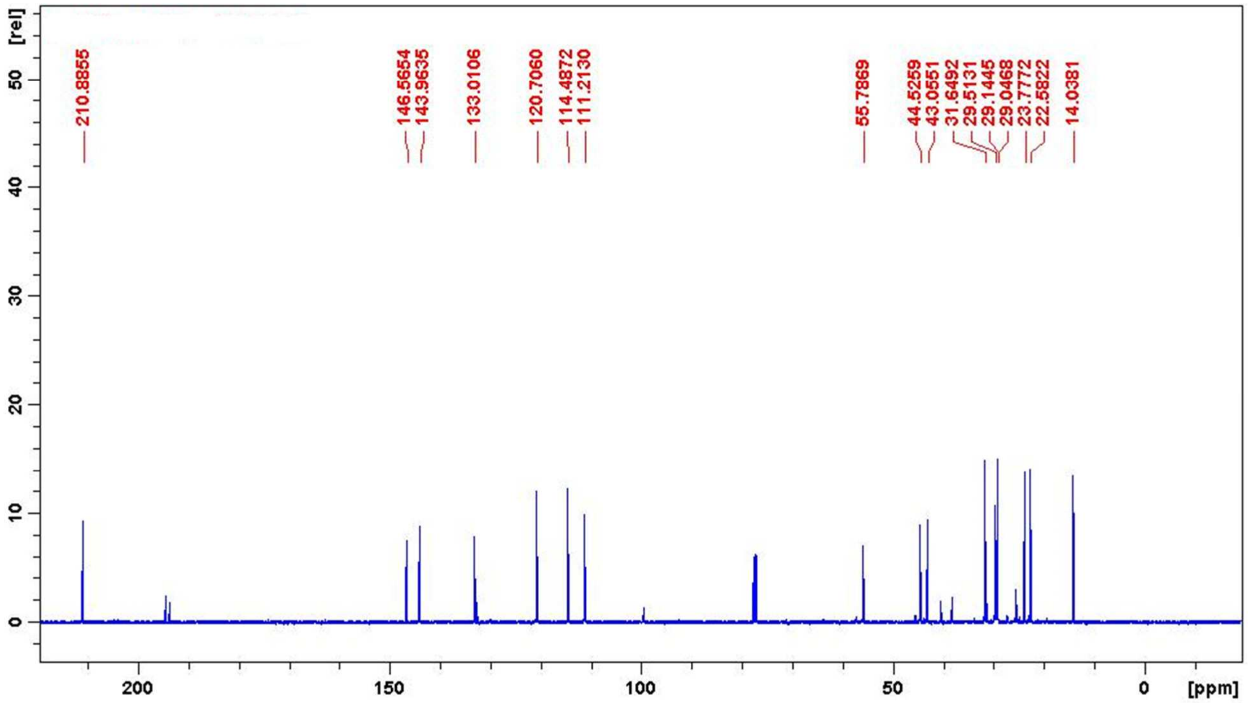

Figure S2: ^{13}C NMR spectrum of 6-paradol (**1**) ($CDCl_3$, 400 MHz).

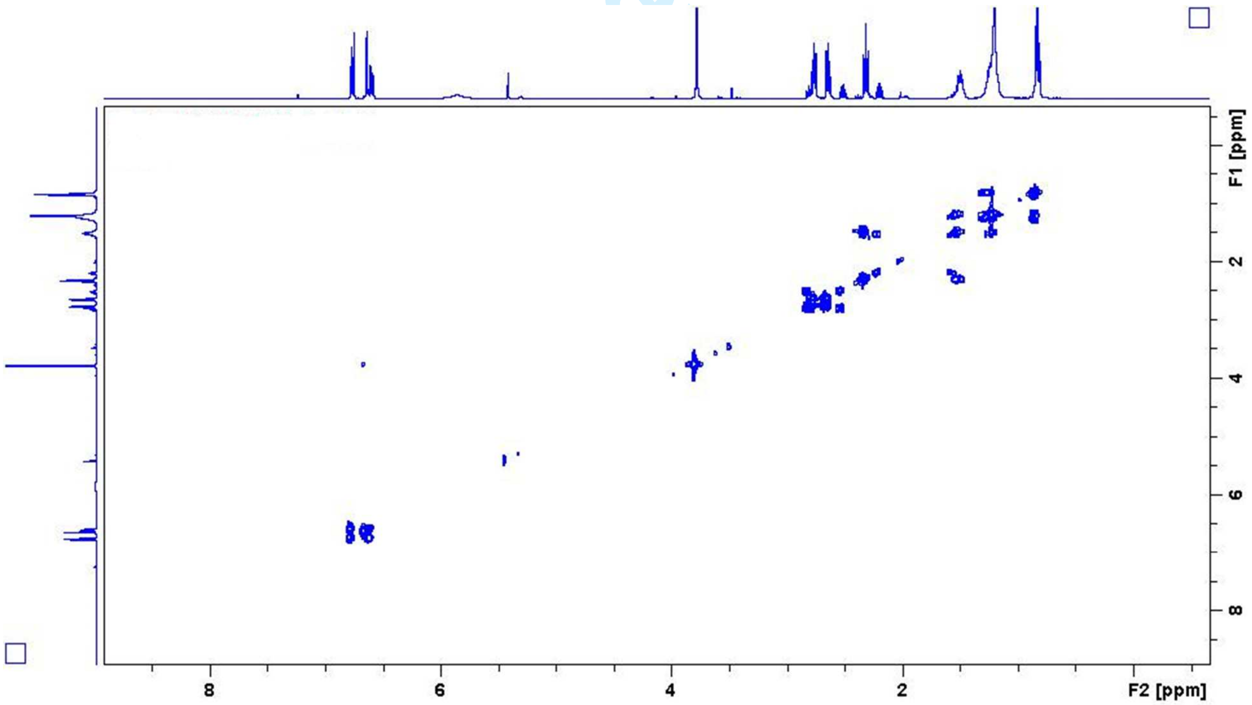

Figure S3: COSY NMR spectrum of 6-paradol (**1**)

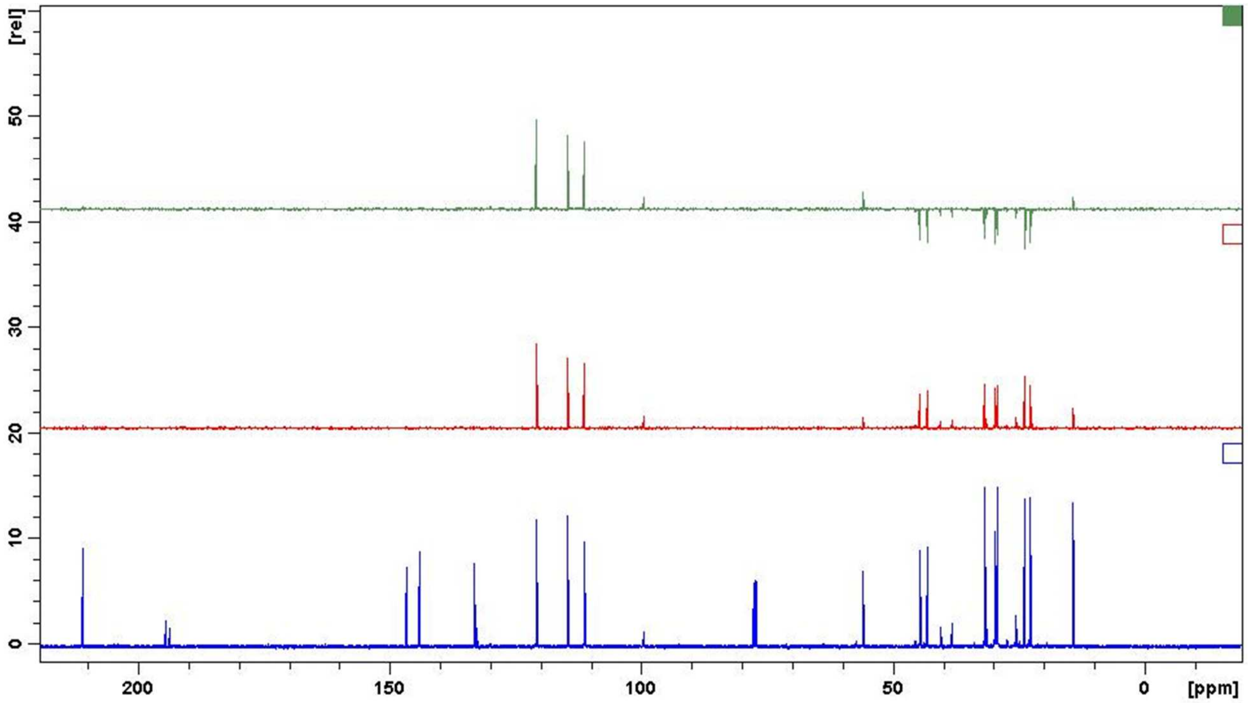

Figure S4: DEPT spectrum of 6-paradol (**1**)

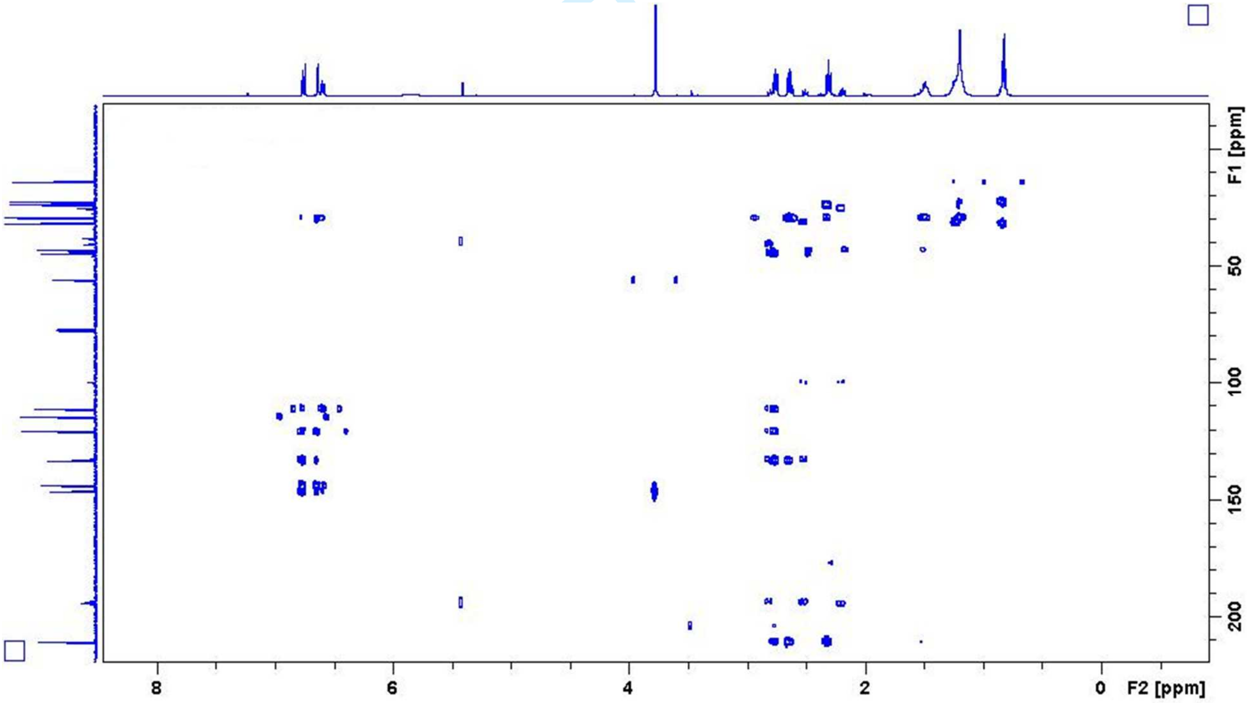

Figure S5: HMBC NMR spectrum of 6-paradol (**1**)

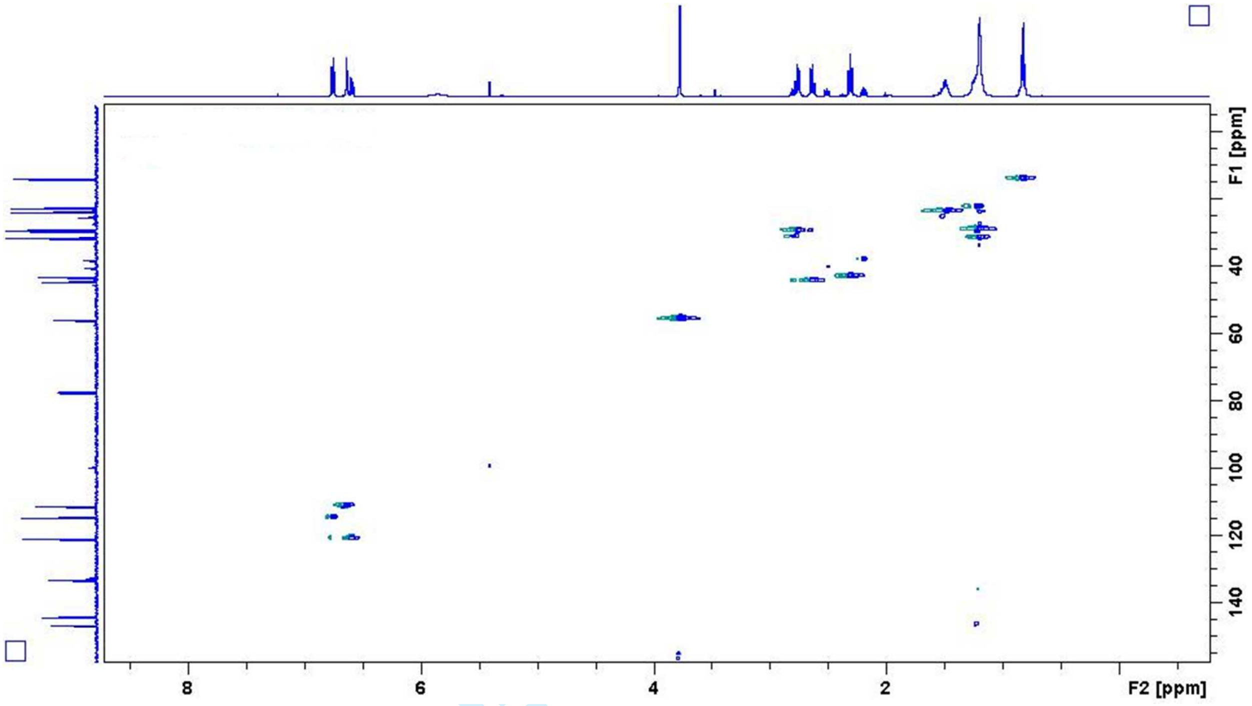

Figure S6: HSQC NMR spectrum of 6-paradol (**1**)

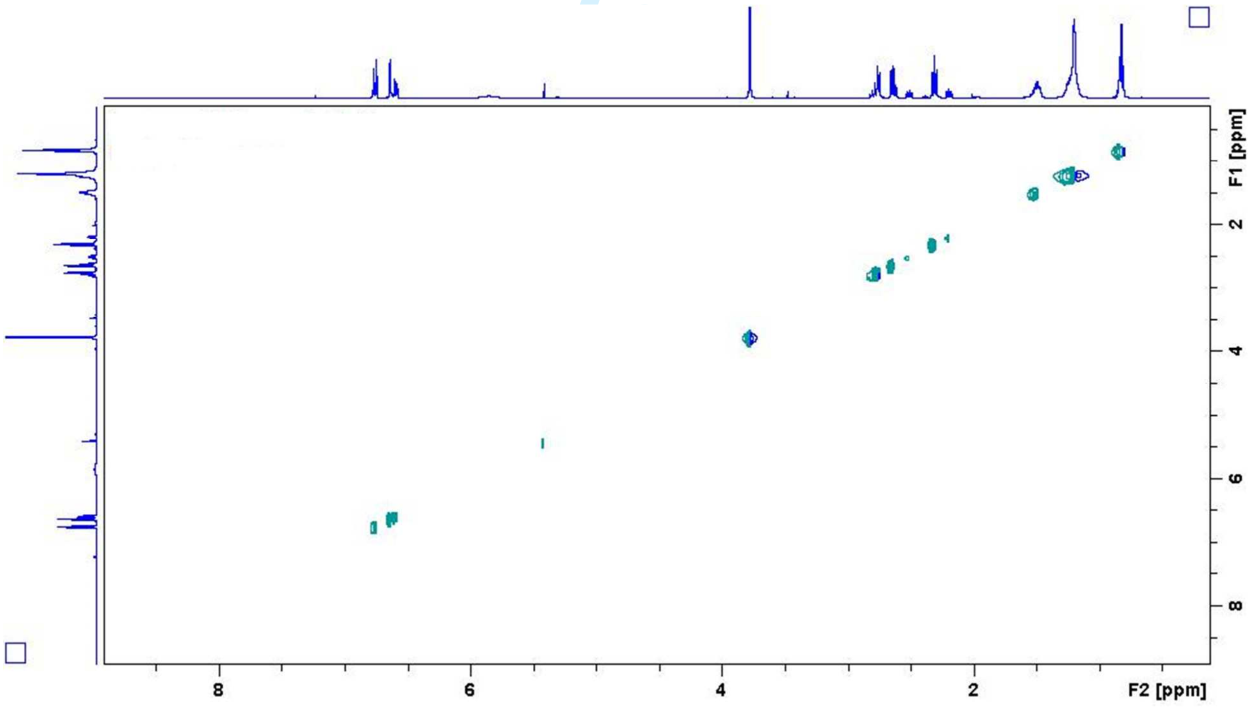

Figure S7: NOESY NMR spectrum 6-paradol (**1**)

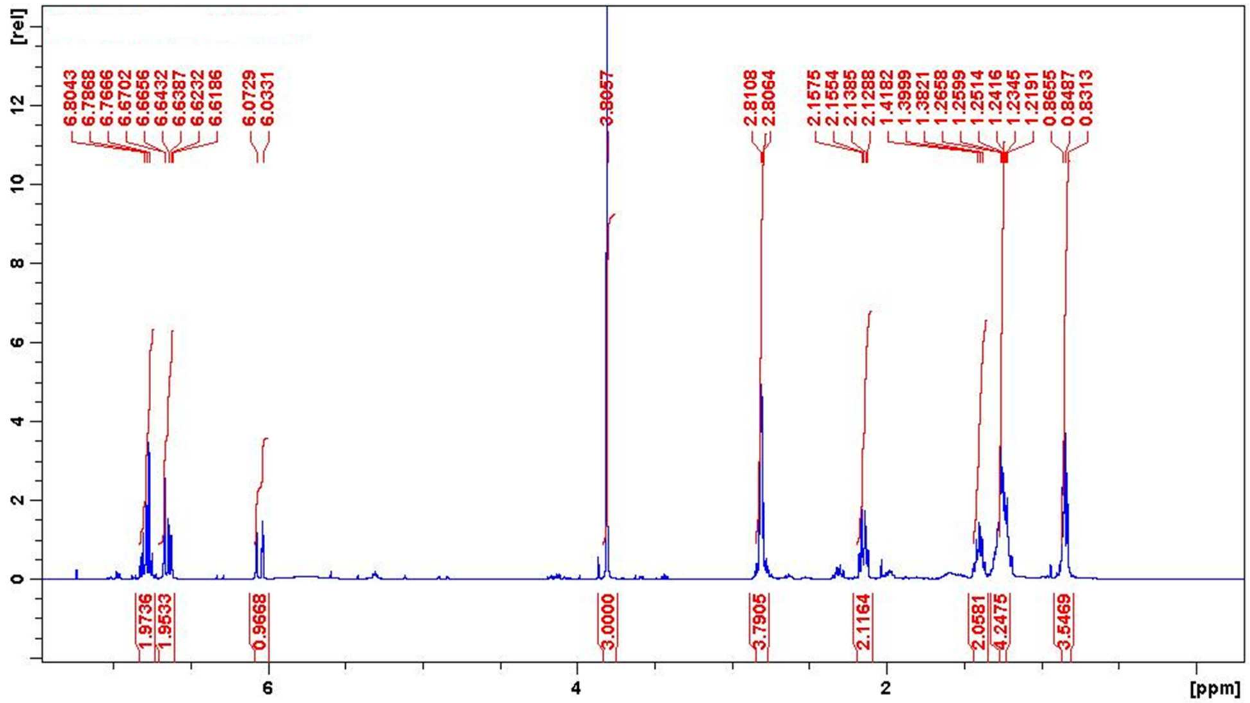

Figure S8: ^{1}H NMR spectrum of 6-shagaol (**2**) ($CDCl_3$, 400 MHz).

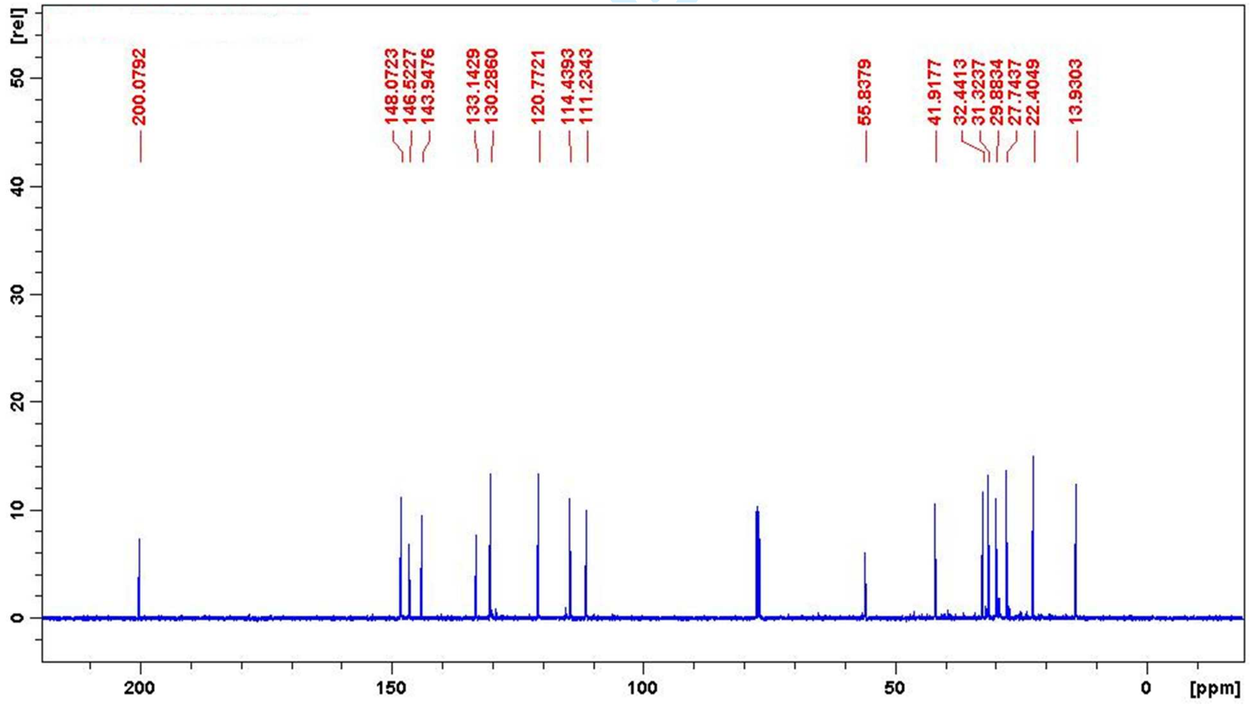

Figure S9: ^{13}C NMR spectrum of 6-shagaol (**2**)

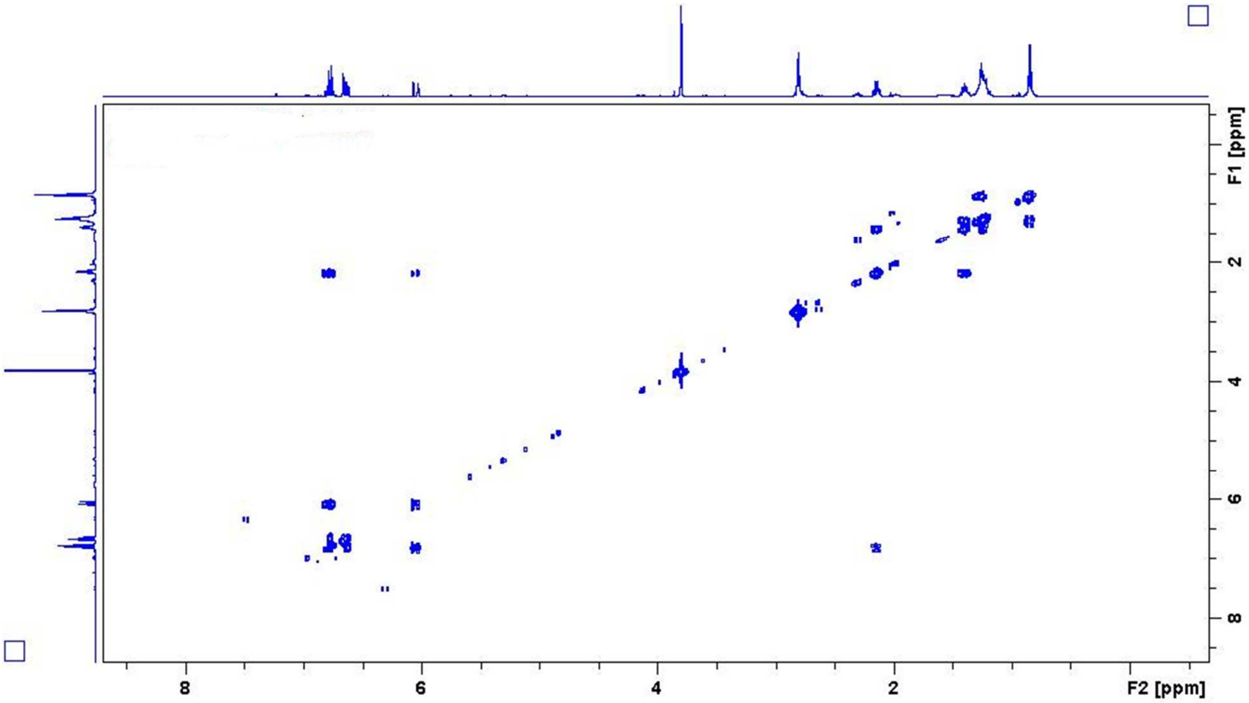

Figure S10: COSY NMR spectrum of 6-shagaol (**2**)

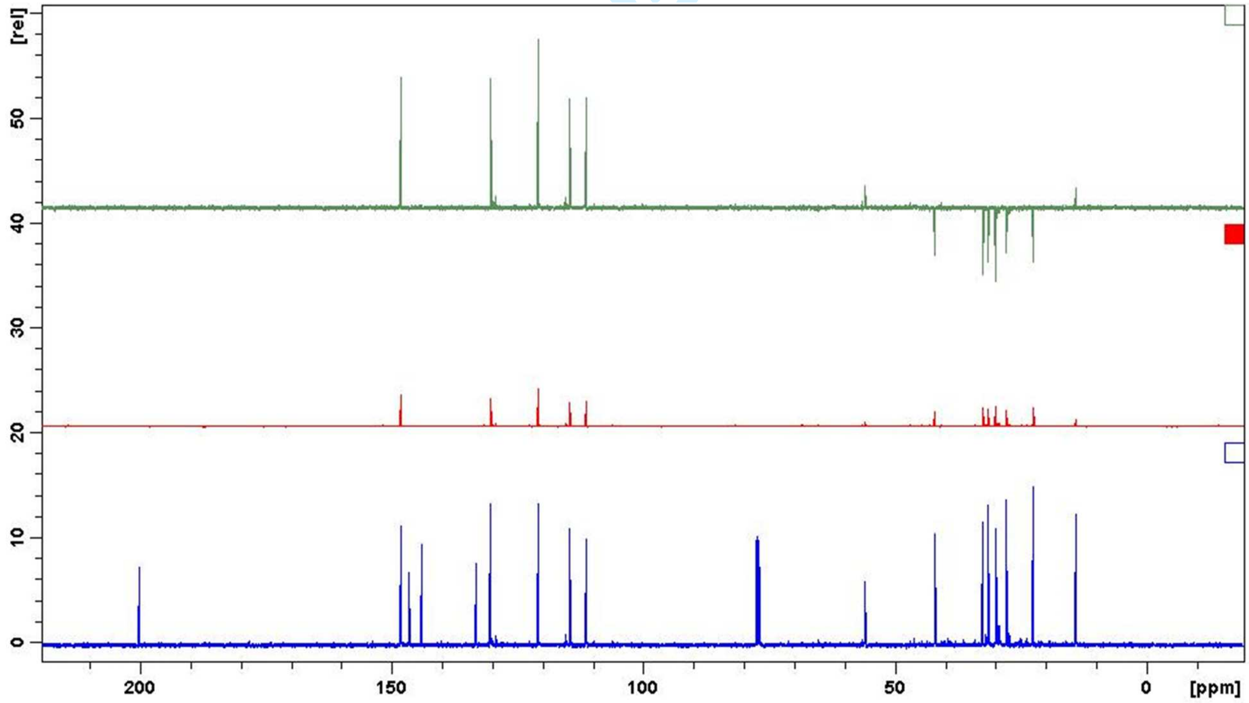

Figure S11: DEPT spectrum of 6-shagaol (**2**)

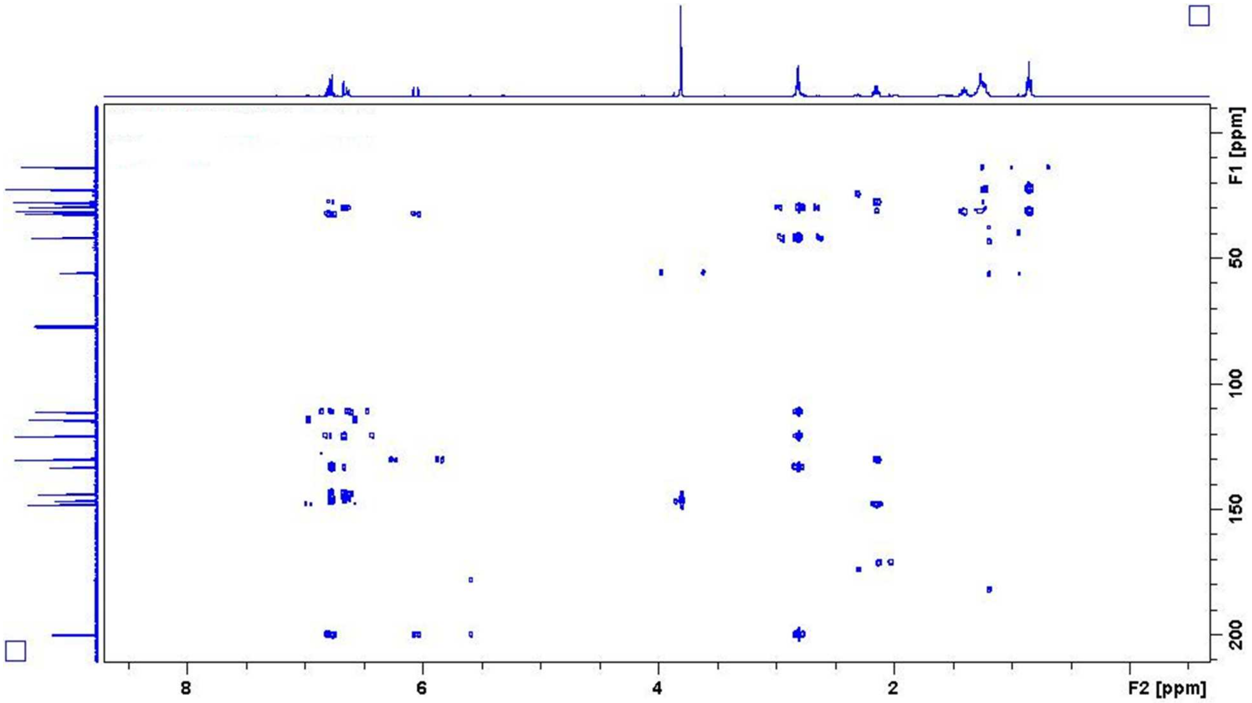

Figure S12: HMBC NMR spectrum of 6-shagaol (**2**).

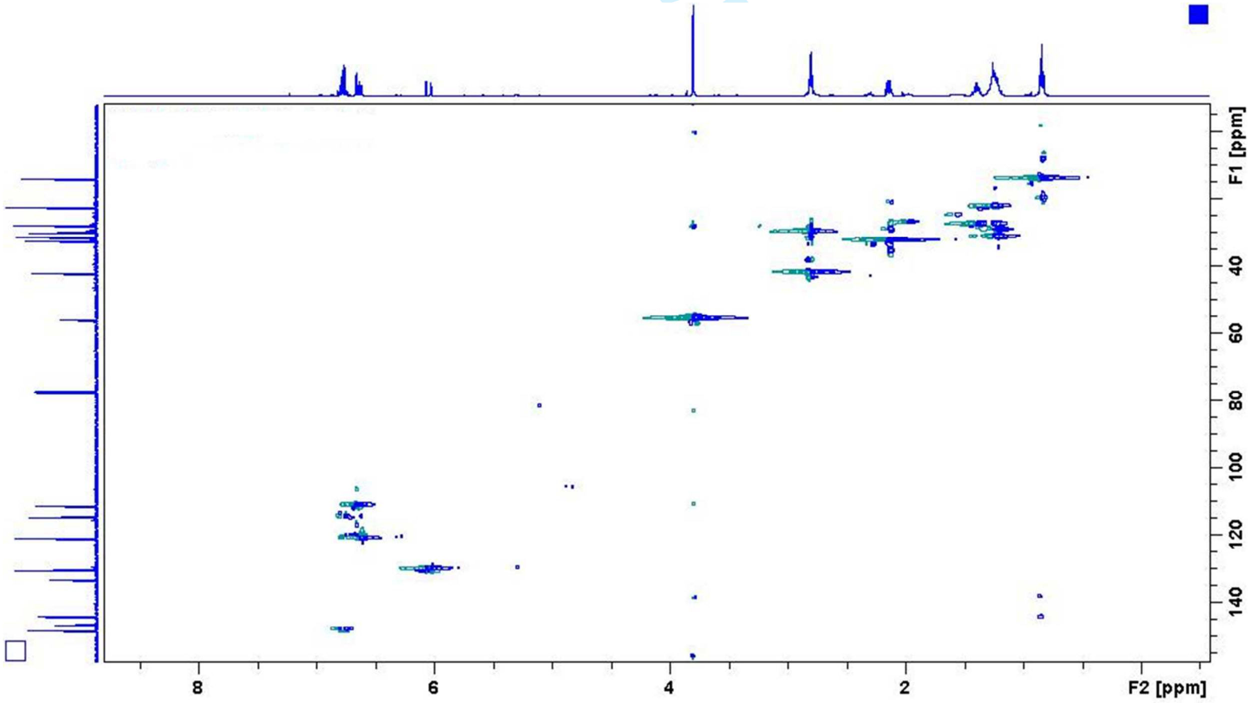

Figure S13: HSQC NMR spectrum of 6-shagaol (**2**).

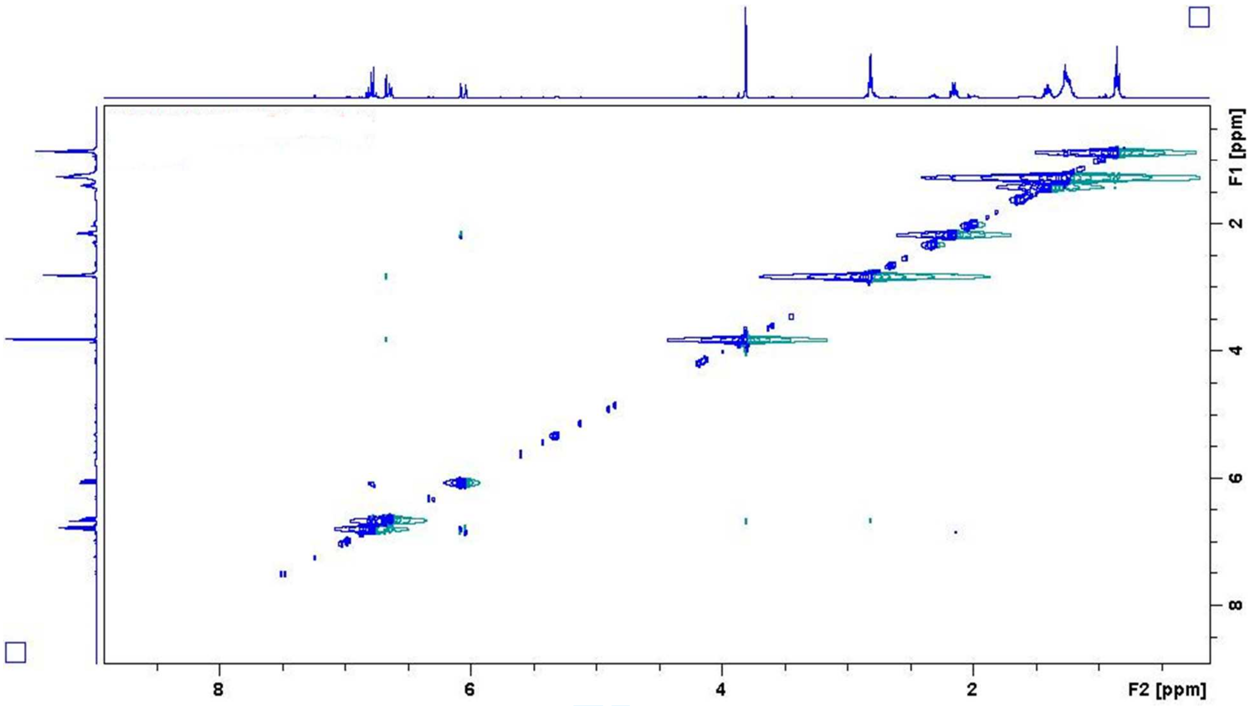

Figure S14: NOESY NMR spectrum 6-shagaol (**2**).

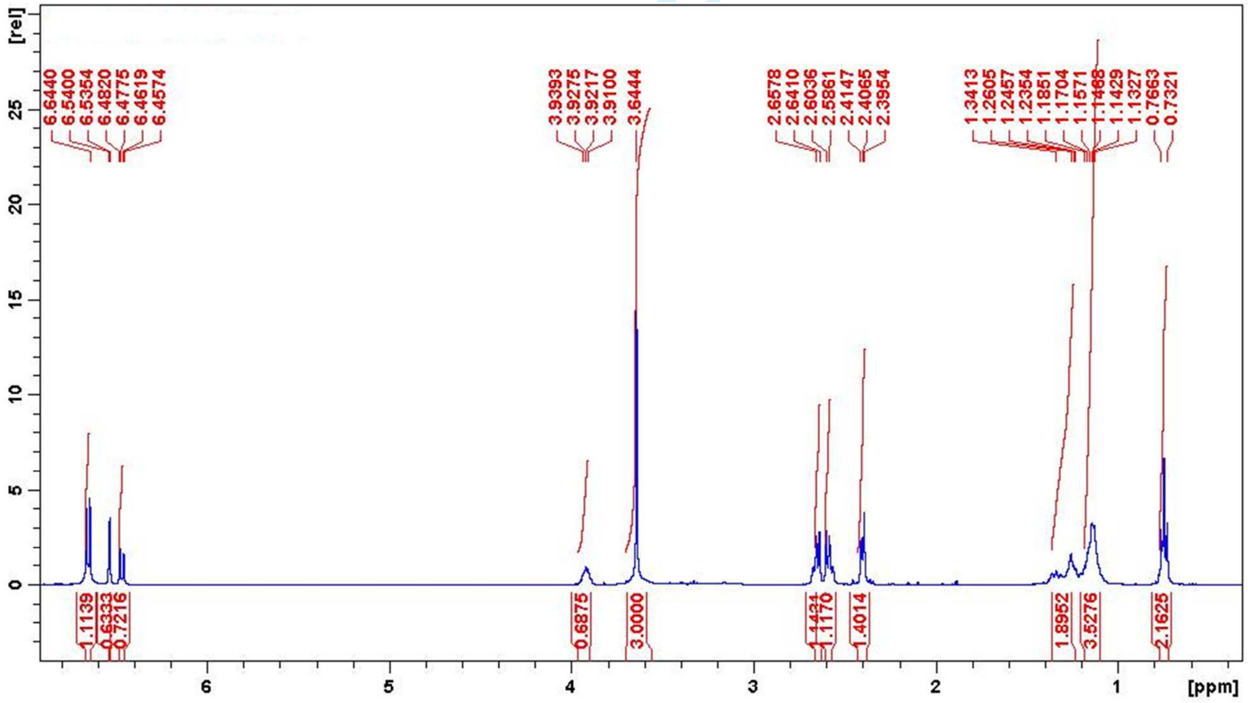

Figure S15: ^{1}H NMR spectrum of 6-gingerol (**3**)

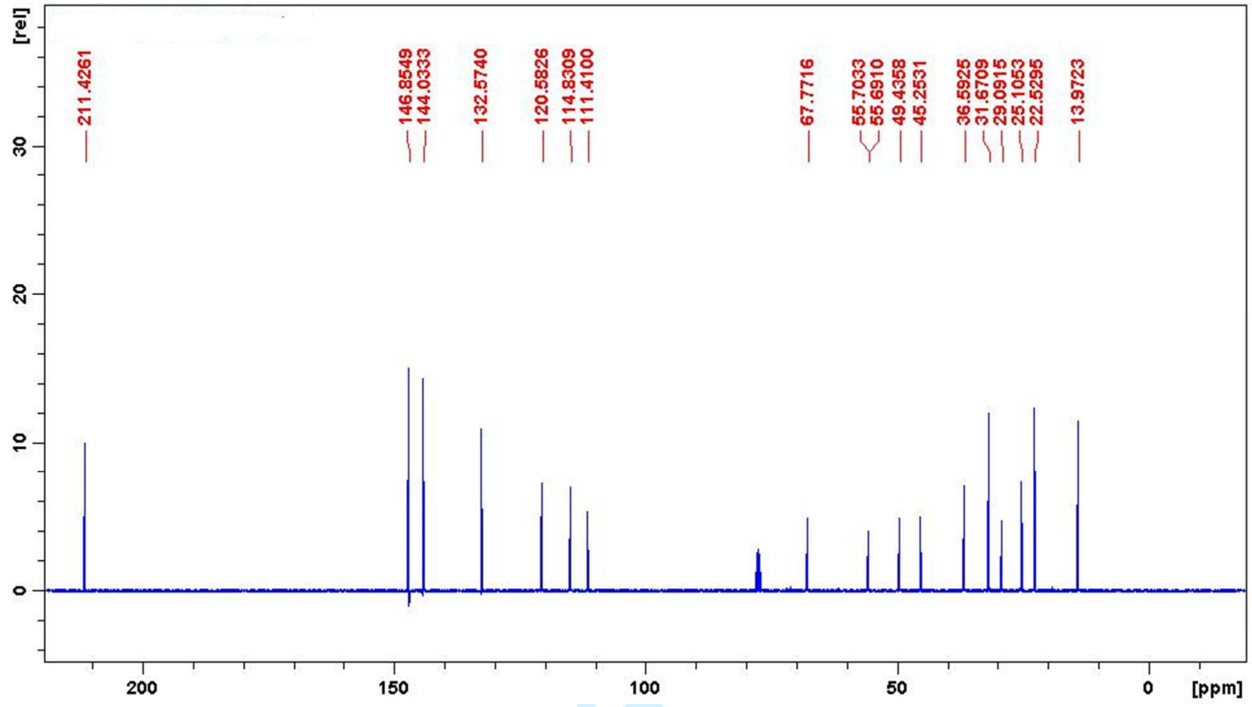

Figure S16: ^{13}C NMR spectrum of 6-gingerol (**3**).

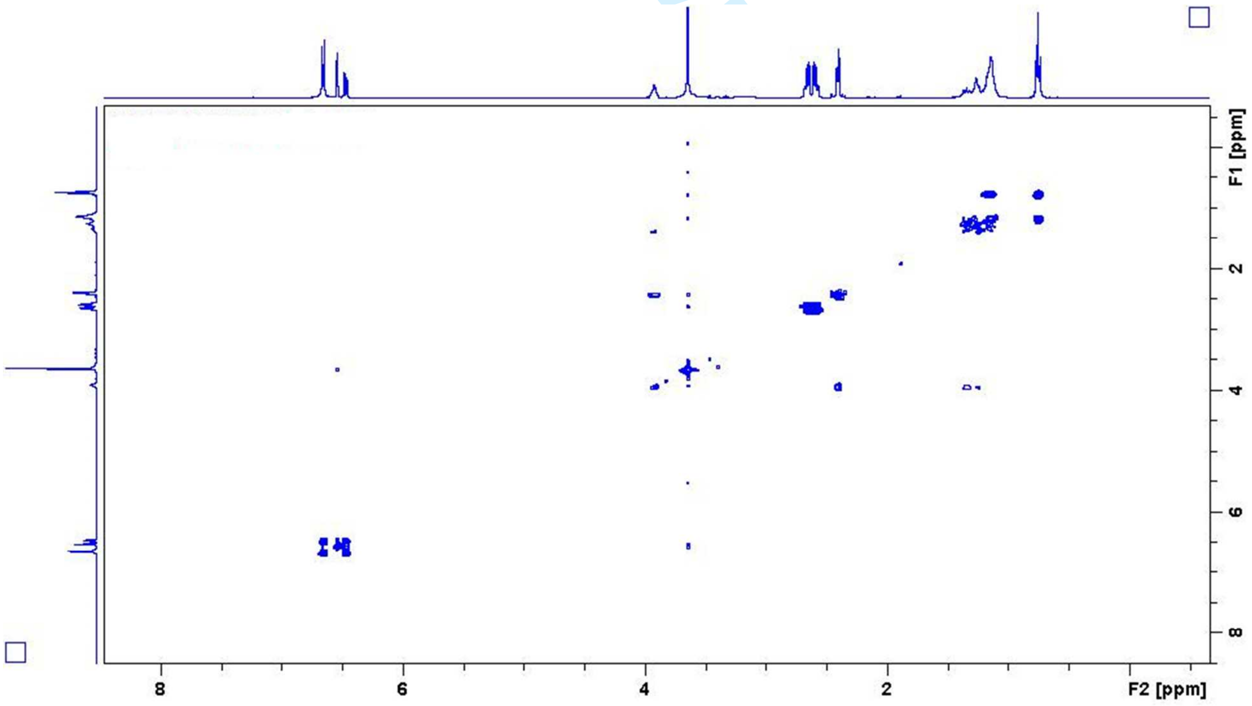

Figure S17: COSY NMR spectrum of 6-gingerol (**3**)

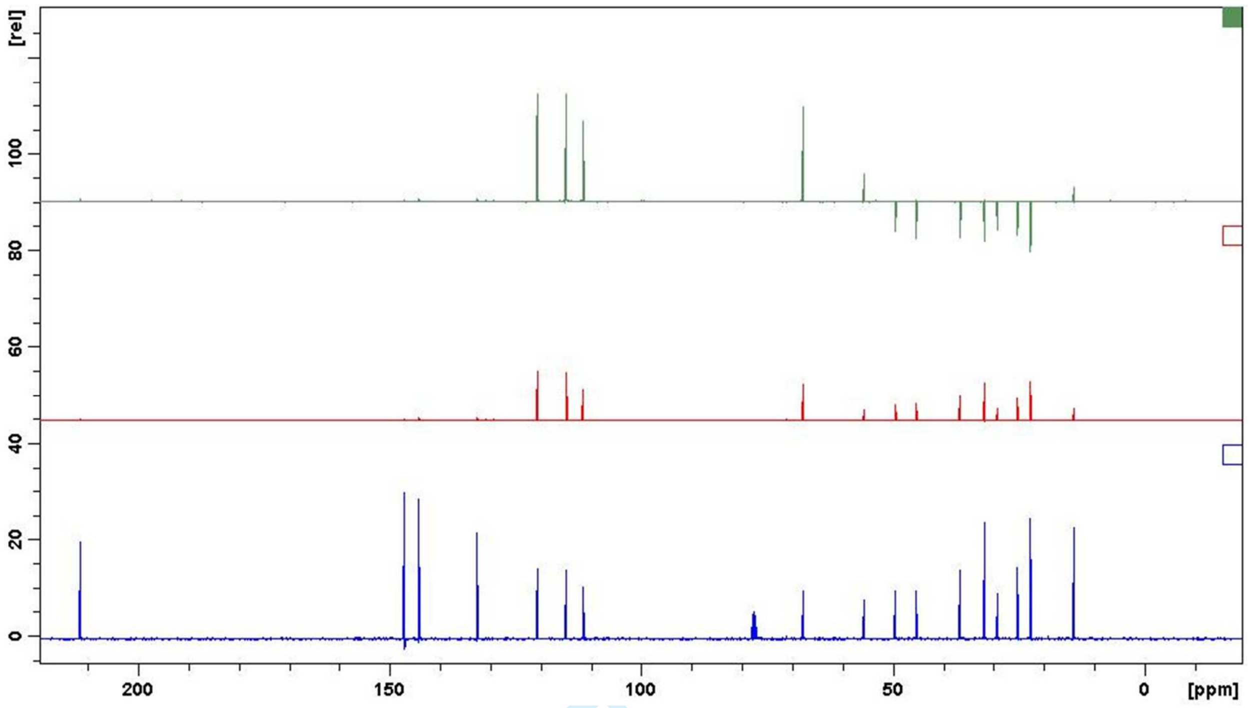

Figure S18: DEPT spectrum of 6-gingerol (**3**)

Revi

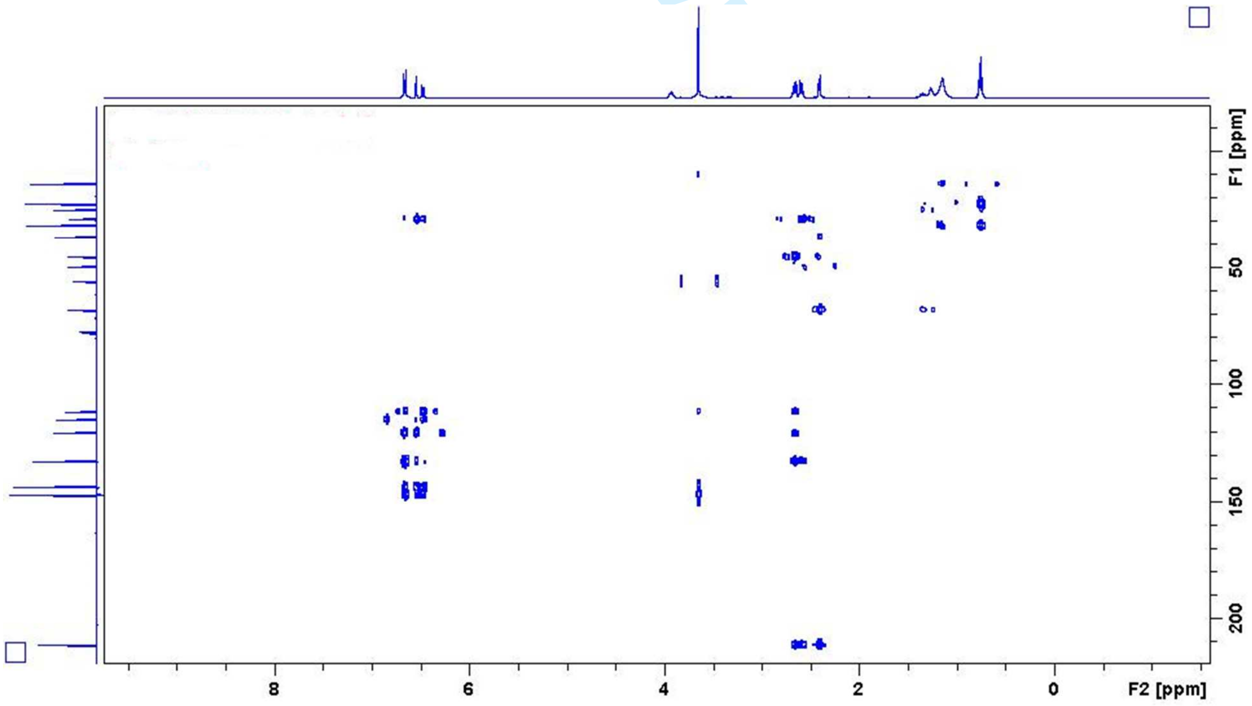

Figure S19: HMBC NMR spectrum of 6-gingerol (**3**).

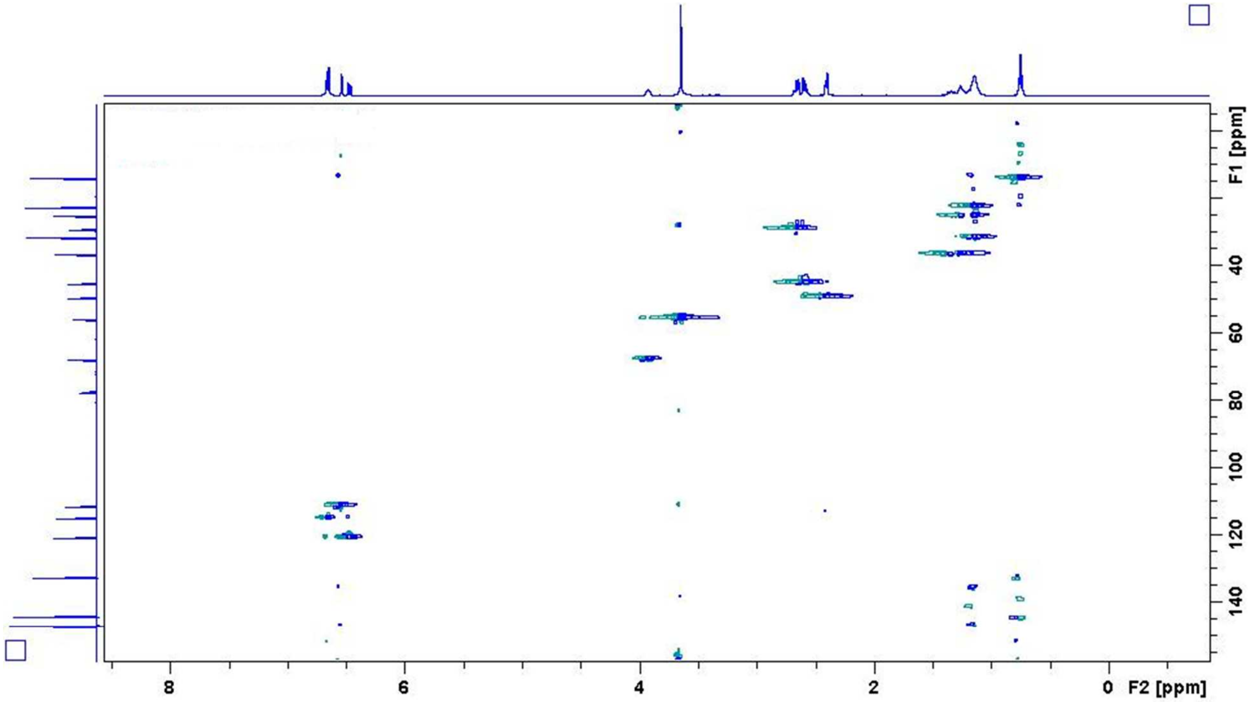

Figure S20: HSQC NMR spectrum of 6-gingerol (**3**).

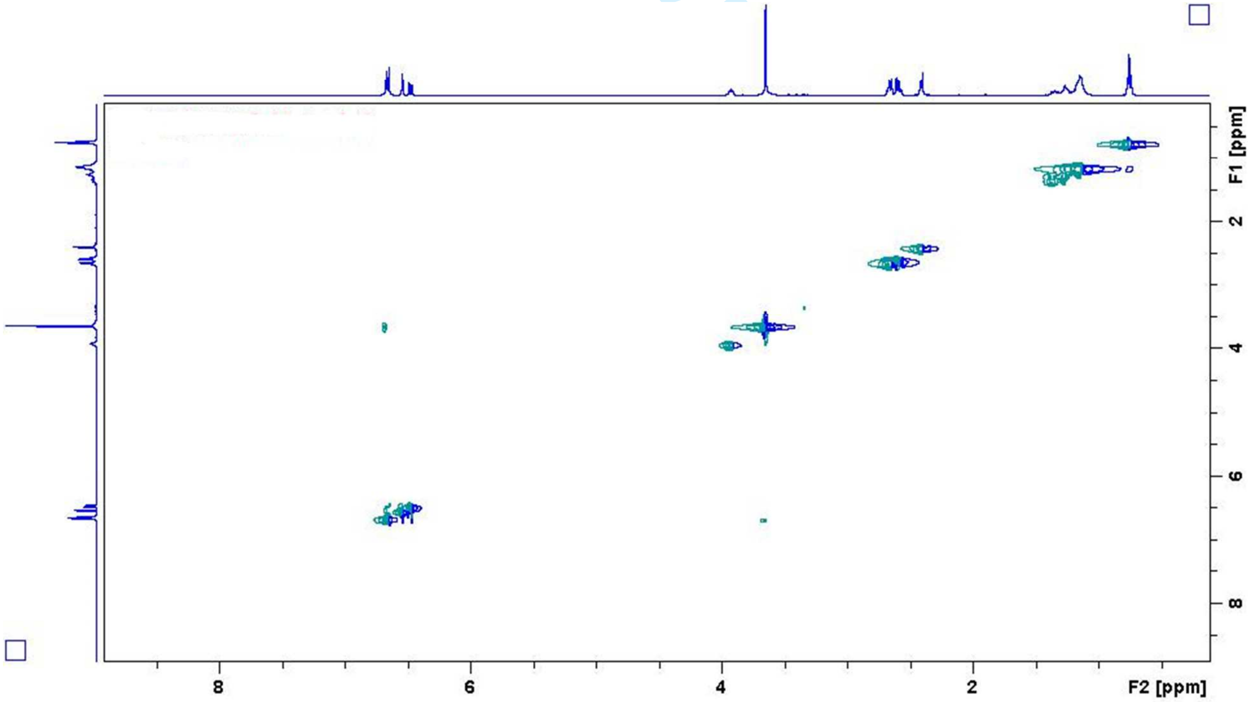

Figure S21: NOESY NMR spectrum 6-gingerol (**3**).

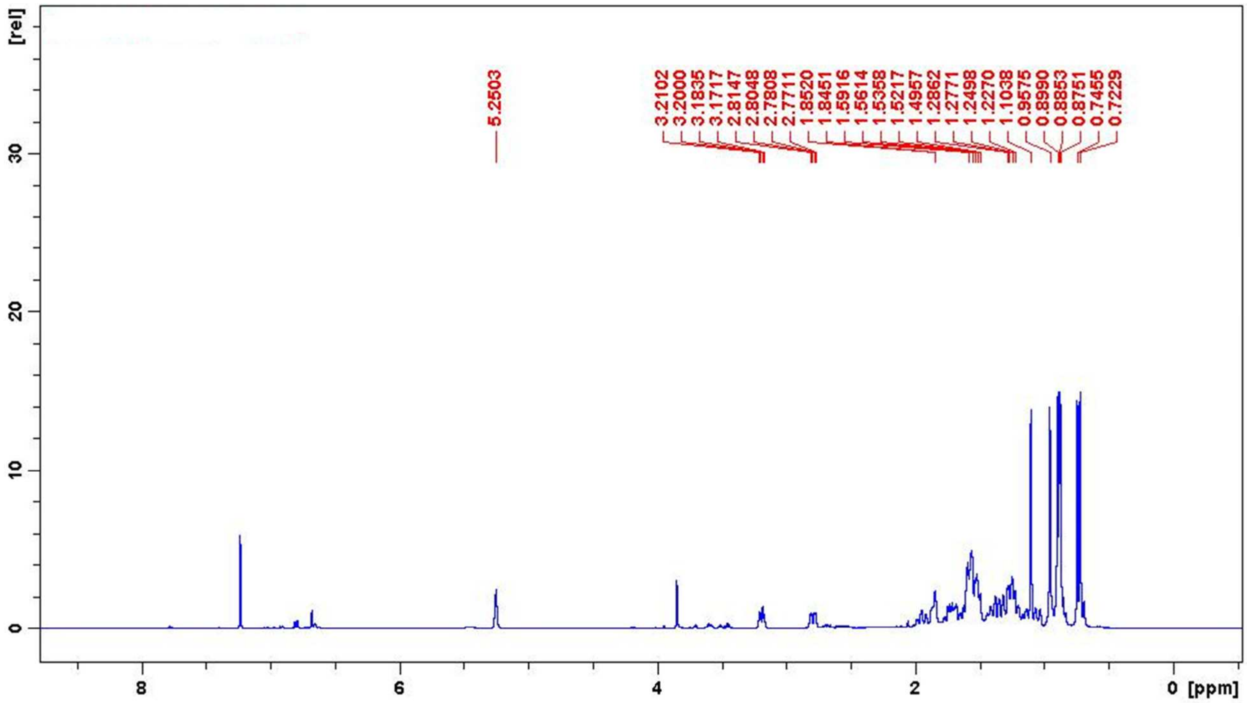

Figure S22: ^{1}H NMR spectrum of oleanolic acid (**4**)

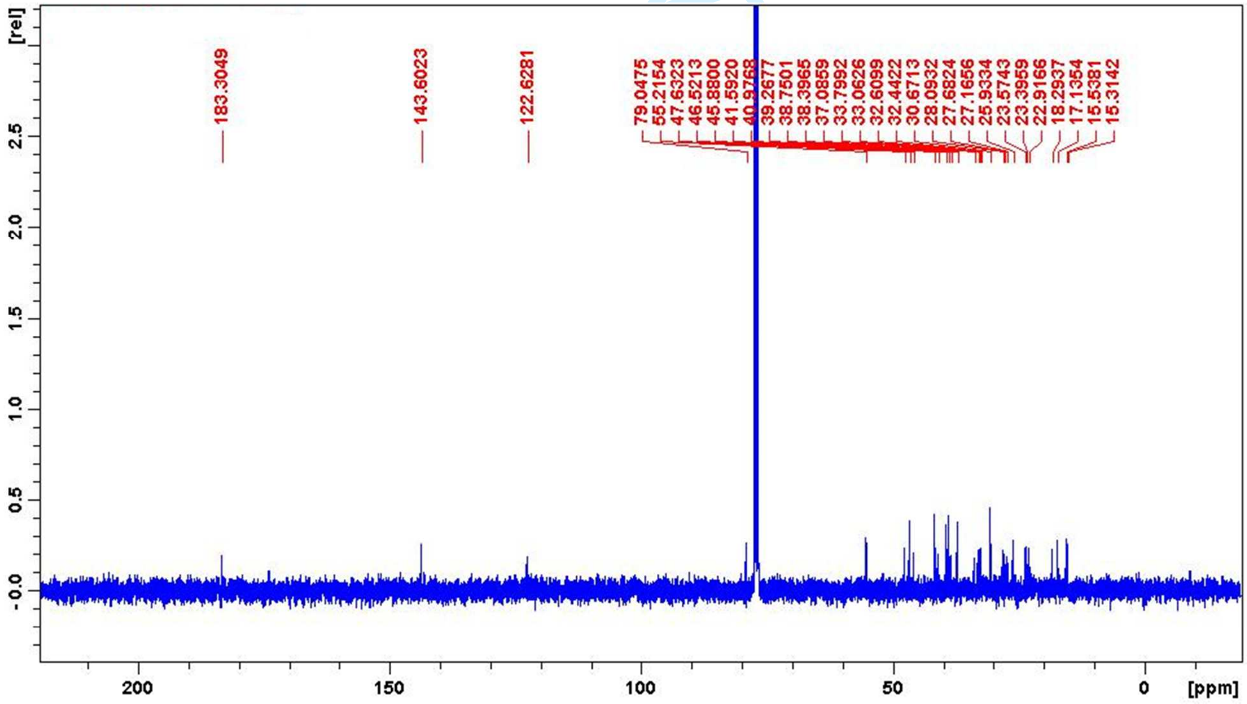

Figure S23: ^{13}C NMR spectrum of oleanolic acid (**4**)

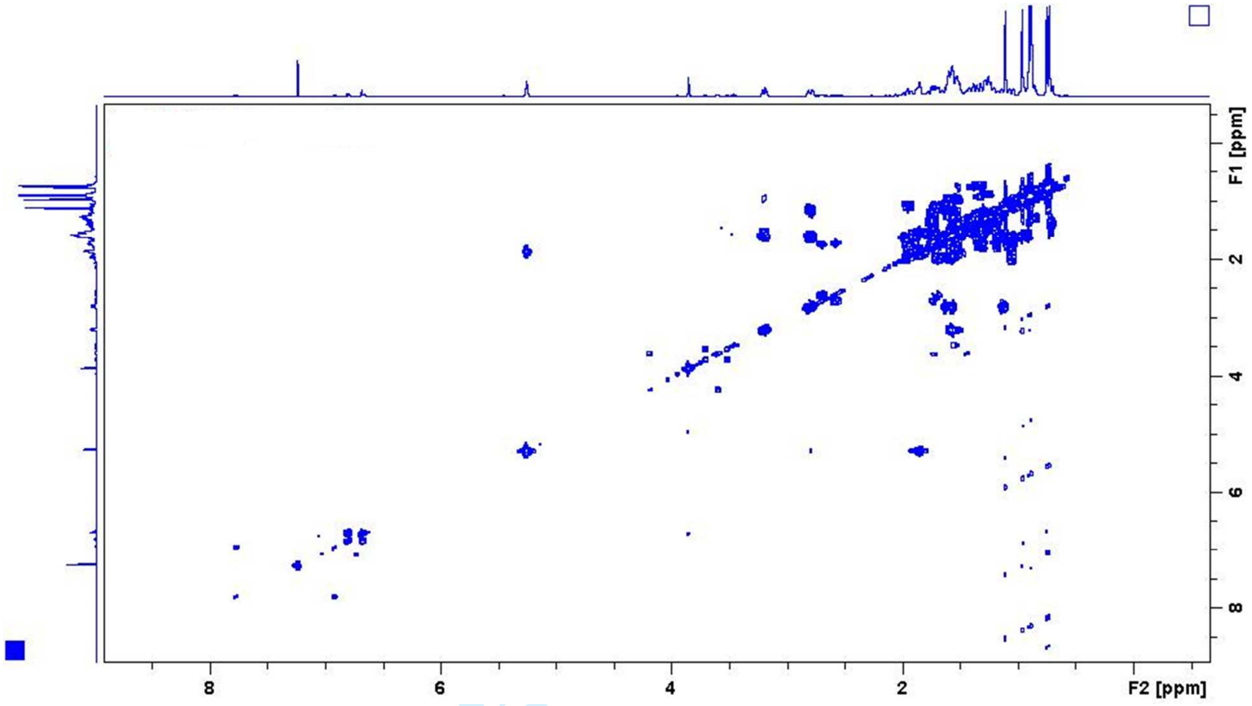

Figure S23: COSY NMR spectrum of oleanolic acid (**4**)

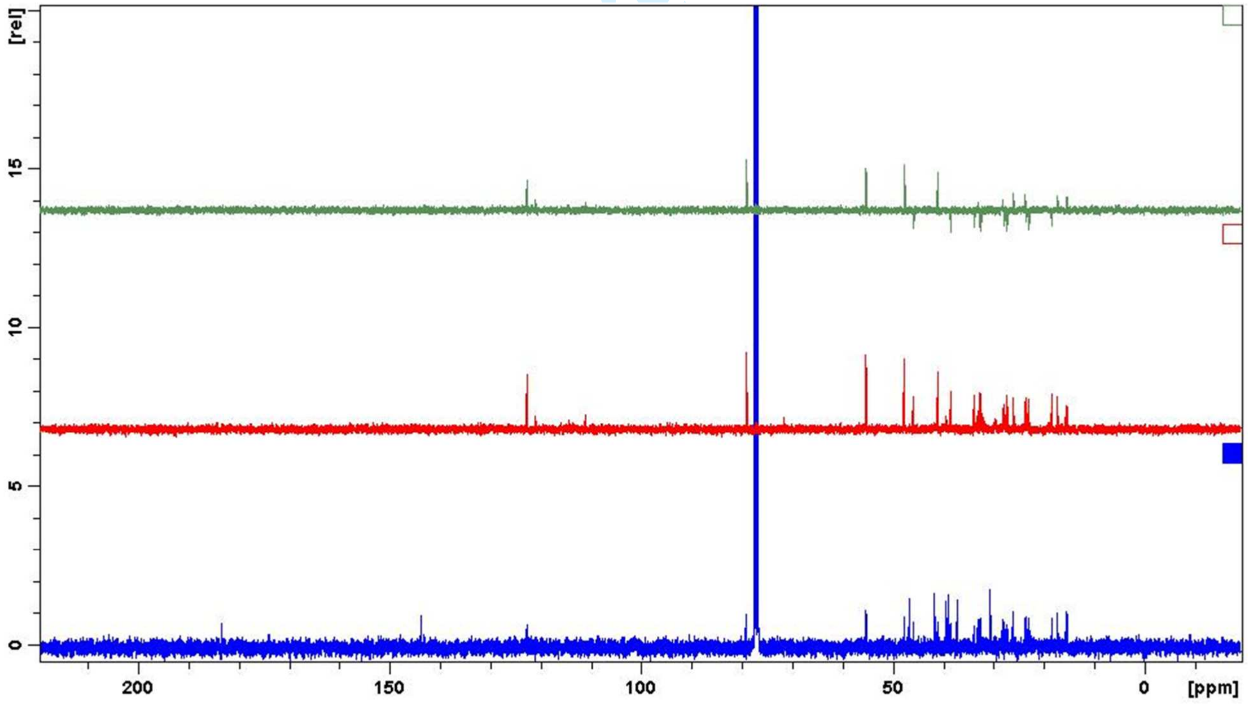

Figure S24: DEPT spectrum of oleanolic acid (**4**)

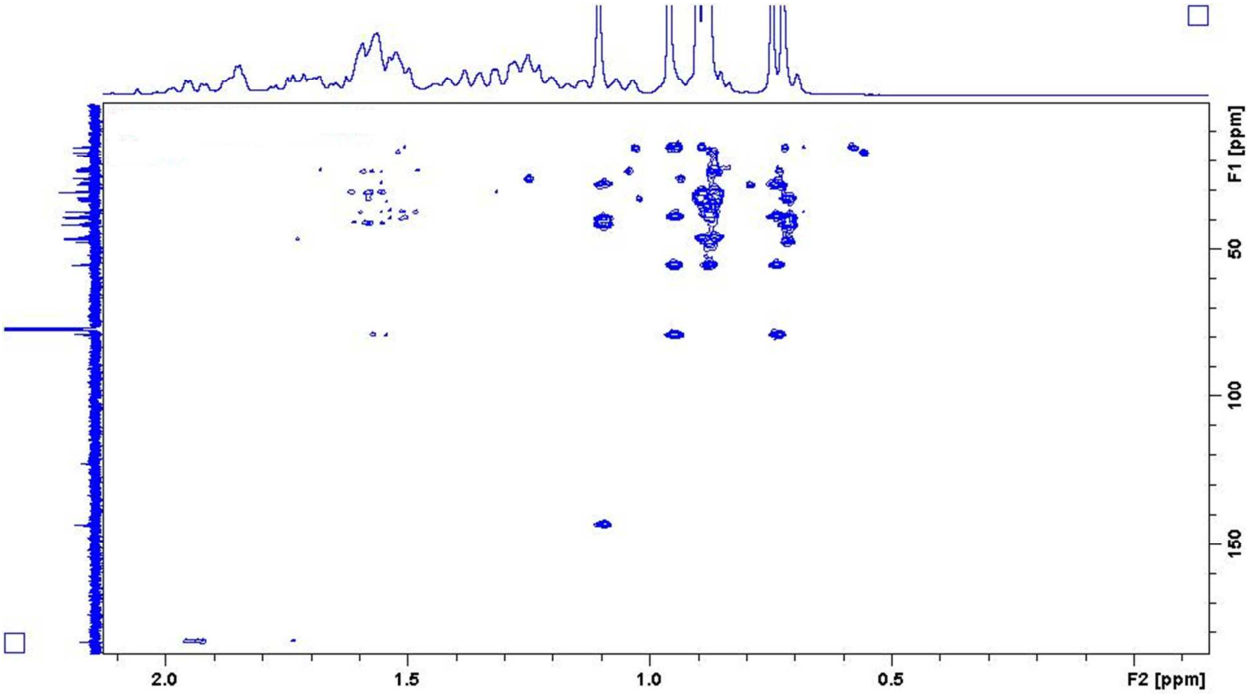

Figure S25: HMBC NMR spectrum of oleanolic acid (**4**).

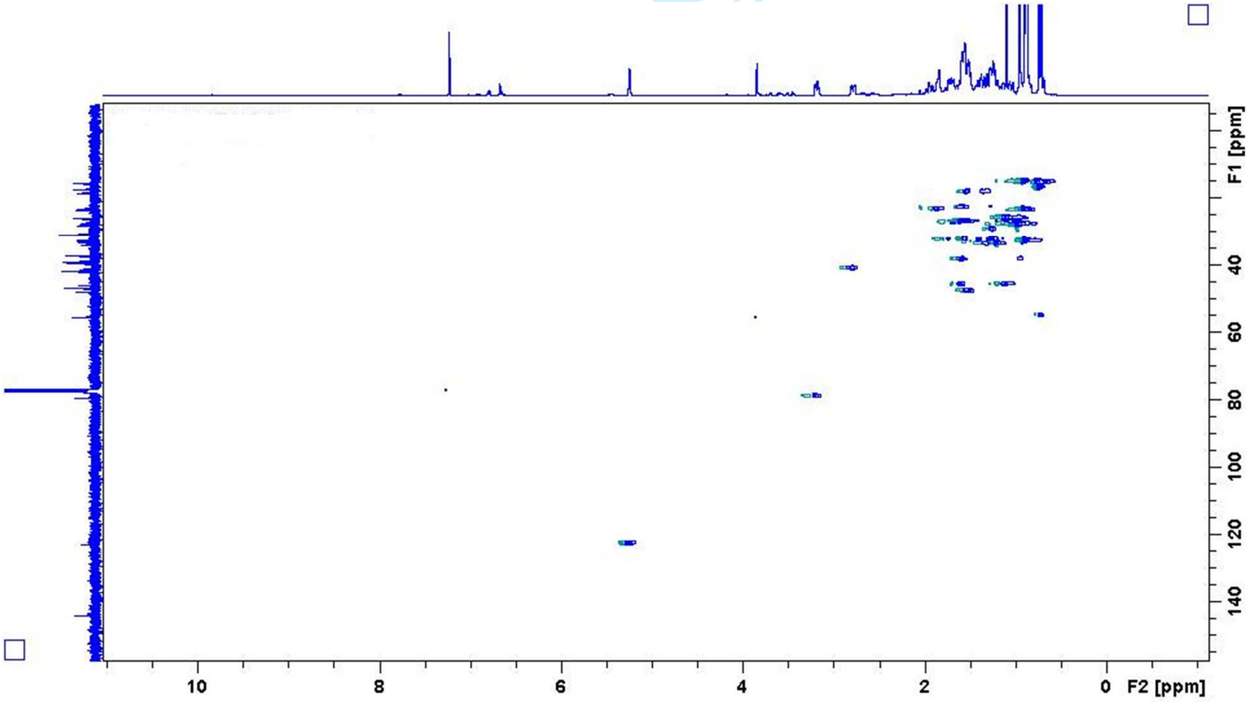

Figure S26: HSQC NMR spectrum of oleanolic acid (**4**)

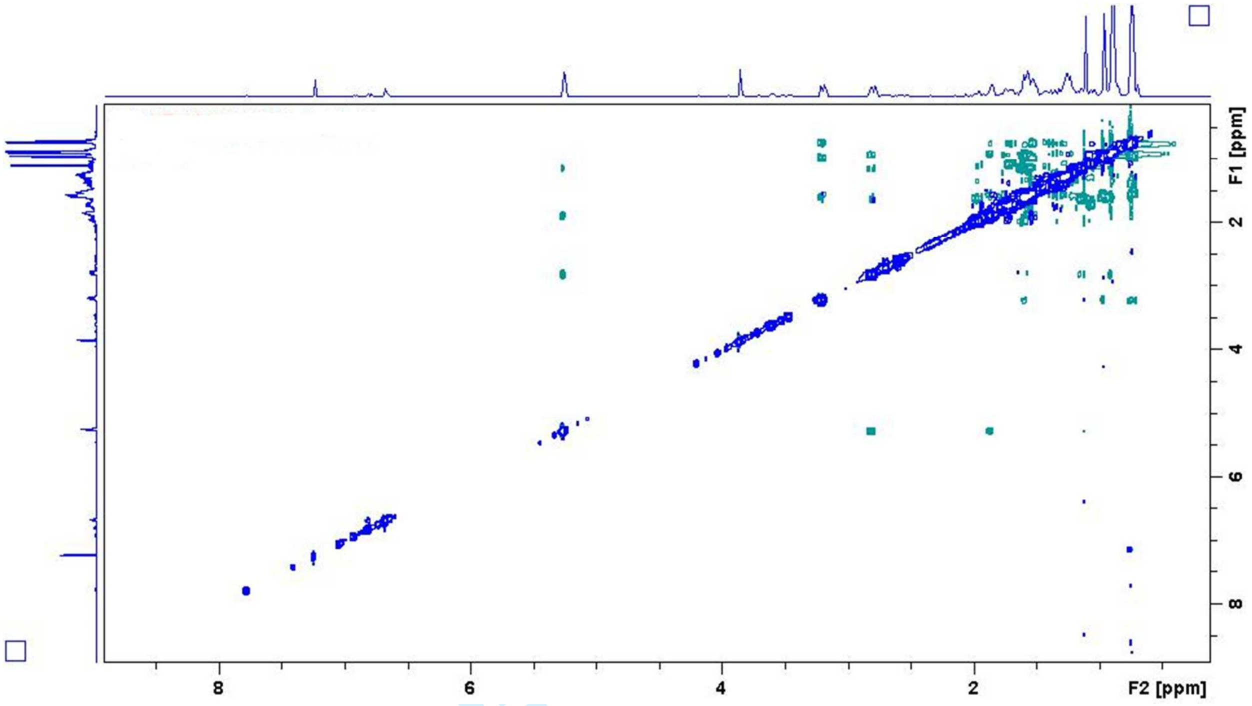

Figure S27: NOESY NMR spectrum oleanolic acid (**4**).

For Peer Review Only
